# Effects of one night of sleep deprivation on single- and dual-task gait

**DOI:** 10.1098/rsif.2026.0047

**Published:** 2026-10-07

**Authors:** Maura Seynaeve, Anna C. Render, Peter C. Fino, Toon de Beukelaar

**Affiliations:** ^1^ Department of Movement Sciences, KU Leuven, Leuven, Belgium; ^2^ KU Leuven Institute of Sports Science, Leuven, Belgium; ^3^ Health & Kinesiology, University of Utah, Salt Lake City, UT, USA

**Keywords:** sleep deprivation, biomechanics, dual-task walking, cognitive-motor interference, foot placement control, margins of stability, spatio-temporal gait parameters, attention

## Abstract

Sleep deprivation (SDEP) impairs cognitive control, which may affect movements that rely on these processes, such as walking. To test whether gait changes after one night of SDEP reflect reduced cognitive capacity, we compared its effects with those of dual-task walking (i.e. walking while performing a simultaneous cognitive task). We hypothesize that SDEP will produce gait changes similar to those under dual-task conditions. Eighteen healthy adults (nine female, nine male; age, mean (s.d.): 22.2 (2.3) years) were tested the morning after a SDEP and a control night. Participants completed two 2-minute trials: single-task walking and walking with a concurrent 2-back working memory task (DT, dual-task walking). Using lateral foot and pelvis marker trajectories, we calculated spatio-temporal parameters, foot placement error in anteroposterior (FPE_AP_) and mediolateral (FPE_ML_) directions, and mediolateral margin of stability (MoS_ML_). SDEP increased average step time (*p* < 0.001) and step length (*p* = 0.002), and DT reduced spatio-temporal variability. Both SDEP (*p* = 0.004) and DT (*p* < 0.001) reduced FPE_AP_, but only DT reduced FPE_ML_ (*p* < 0.001). Additionally, mean MoS_ML_ decreased only in SDEP (*p* = 0.025). Overall, these findings suggest that while SDEP and dual-tasking both affect gait, the effects of SDEP on gait cannot be fully explained by reduced cognitive resources.

## 1. Introduction

Sleep is a biological process essential for maintaining physical health, cognitive function and overall well-being across the lifespan. Besides the well-known effects of sleep loss on cognitive performance [1], there is a growing body of literature showing that sleep loss also affects motor performance [2–6]. In general, these studies report a more profound effect of sleep loss for continuous and multi-joint tasks (e.g. maintaining balance) in comparison with discrete and single-joint tasks (e.g. a maximal knee extension). These continuous, multi-joint tasks rely on neural control processes that exceed basic spinal reflex mechanisms, including feedback and feed-forward processes, executive control and attention [7,8]. Such findings suggest that sleep loss disproportionately affects movements that rely heavily on these cognitive control processes. For instance, multiple studies have shown that sleep loss impairs balance [9,10], a task that relies on these cognitive control processes [11,12]. However, most of these studies focus on static balance tasks, so it remains unclear whether sleep deprivation (SDEP) causes similar impairments during dynamic activities such as walking.

Observational studies support an association between sleep and gait. Individuals who report poor or insufficient sleep (less than 7 hours or poor sleep quality) show reduced gait speed, lower cadence and larger variability in spatio-temporal parameters [13]. Moreover, poor sleep is associated with increased trunk rotation, larger foot strike angles, longer stride length and extended duration in single-leg support or swing phase, which collectively indicate compromised gait stability and efficiency [14–16]. However, the mechanisms underlying these relationships remain unclear because these prior studies have been purely observational and did not directly manipulate sleep.

Two interventional studies have experimentally altered sleep to examine the effects of SDEP on gait, and these studies further support an association between the two. Oliveira *et al.* [17] demonstrated that SDEP reduces gait complexity, as reflected by a decrease in detrended fluctuation analysis of inter-stride intervals. Thereby, the authors suggest SDEP leads to altered neuromuscular control. In addition, Umemura *et al.* [18] reported that sleep-deprived participants exhibited impaired performance in a sensorimotor synchronization gait task, in which they were required to match their footfalls to auditory cues. Although these studies offer valuable insight into the sensorimotor mechanisms underlying gait, an alternative explanation for these relationships is a change in cognitive reserve after SDEP.

Given the well-established finding that SDEP severely depletes cognitive reserves [1], it is possible that sleep-related alterations in gait stem from a decline in cognitive resources. One method to examine the contribution of these cognitive resources during gait is by introducing a dual-task interference paradigm [19], where a cognitive task and a motor task are performed at the same time. Dual-tasking challenges the central nervous system's ability to manage multiple demands and typically changes the task performance of one or both tasks. This interference can be explained by two distinct theoretical frameworks: bottleneck theory, which suggests that central processing for the second task is delayed until the first task is completed, or capacity-sharing theory, which proposes that central processing for both tasks can occur in parallel but must share limited cognitive resources. Similar to the association between poor sleep and gait, dual-tasking typically results in slower gait speeds and/or an increase in gait variability [20]. Therefore, the observed associations between gait disturbances and sleep might not be purely related to the motor system. Instead, gait alterations after insufficient sleep may represent the central nervous system's difficulty in allocating sufficient cognitive resources to maintain normal gait.

To test this hypothesis, the present study aimed to examine the effects of one night of SDEP on gait control and stability in single- and dual-task walking conditions. Anteroposterior (AP) gait dynamics are largely governed by passive pendular mechanics and momentum, whereas mediolateral (ML) gait stability requires more active, continuous neural regulation to counteract the inherently unstable ML dynamics of bipedal locomotion [21–23]. Therefore, we separately examined foot placement control and gait stability in both the AP and ML directions, as well as broader spatio-temporal parameters, to capture the full spectrum of gait adaptations under each condition. Using a randomized, crossover design, participants underwent two testing sessions: one following a normal night of sleep (CON, control) and one following 24 hours of total SDEP. We hypothesized that SDEP would produce effects similar to those observed under dual-task gait conditions, reflecting a shared underlying mechanism of reduced cognitive resource availability. In addition, we predicted that the combination of SDEP and dual-tasking would further exacerbate changes in gait parameters.

## 2. Methodology

### 2.1. Participants

Twenty-two healthy adults aged 18–35 years were recruited for the study. Sleep quality and daytime sleepiness were evaluated using the Pittsburgh Sleep Quality Index (PSQI) and the Epworth Sleepiness Scale (ESS), respectively. Only participants with a PSQI score less than or equal to 5 and an ESS score less than 11 were included to ensure adequate baseline sleep quality. Individuals with suspected or diagnosed sleep disorders were excluded. Additional exclusion criteria comprised daily alcohol consumption, caffeine intake exceeding 400 mg day^−1^, use of psychoactive substances, active smoking and any known metabolic, cardiovascular, respiratory or orthopaedic conditions that could influence task performance or sleep. Because a healthy sleep duration is defined as more than 7 hours, participants who slept less than 7 hours in the control condition were excluded. Based on this criterion, we had to exclude four participants, leaving 18 participants (nine female, nine male; age, mean (s.d.) = 22.2 (2.3) years; weight 66.8 (9.1) kg; height 1.74 (8.96) m) for data analysis. The study was approved by the Ethics Committee of UZ Leuven (S67790) and conducted in accordance with the Declaration of Helsinki. All participants provided written informed consent.

### 2.2. General study design

Seven days before the first experimental session, participants completed a habituation session during which they (i) slept in the laboratory to become accustomed to the sleep environment and (ii) were fully acquainted with the cognitive and motor tasks (figure 1A). In the 7 days before each of the two experimental sessions, participants were asked to maintain their normal sleep habits. During this period, physical activity and sleep were monitored using wrist-worn ActiGraph wGT3X-BT accelerometers (ActiGraph LLC, Pensacola, FL, USA) and the consensus sleep diary [24]. Participants were instructed to abstain from alcohol and caffeine before each experimental session.

**Figure 1.**
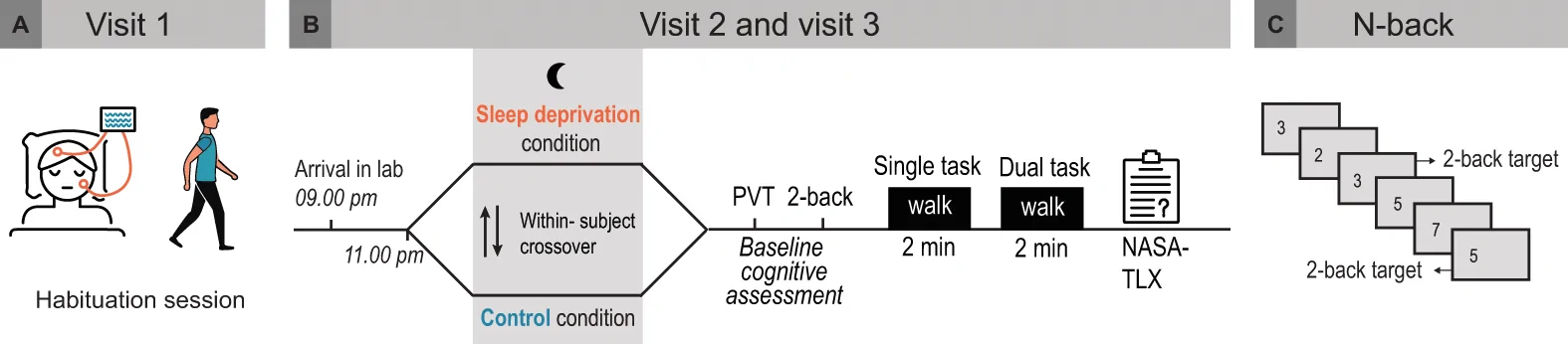
Study design and N-back task procedure. (A) Participants first completed a habituation session (visit 1) to familiarize themselves with the sleep laboratory, polysomnography set-up and motor/cognitive tasks. (B) In two subsequent experimental sessions (visits 2 and 3), participants either stayed awake overnight (sleep deprivation) or slept in the laboratory (control), in a randomized order. In the morning, baseline cognitive assessments included a psychomotor vigilance task (PVT) and a 2-back working memory task. Then, participants completed two 2-minute treadmill trials: normal walking and walking while performing the 2-back task, followed by the NASA Task Load Index (NASA-TLX) questionnaire to assess subjective task load. (C) In the 2-back task, numbers 0–9 were presented, and participants responded when the current number matched the one presented two steps earlier.

Following the habituation session, participants completed two experimental sessions using a randomized, counterbalanced crossover design: one following a night of normal sleep (control condition, CON) and one following a night of total SDEP (figure 1B). Both conditions were interspersed by a one-week wash-out period. During the CON condition, participants slept in the laboratory from 23.00 to 7.00, with full polysomnographic (PSG) monitoring. In the SDEP condition, participants remained awake under continuous supervision throughout the night. They were seated in a dimly lit room (less than 30 lux) and allowed to engage in quiet activities such as reading or solving puzzles, with screen time limited to a maximum of 2 hours in total. Water was provided hourly, and small snacks (e.g. apple, yoghurt or biscuit) were offered at 23.00, 24.00, 1.00 and 2.0. The following morning, participants refrained from food intake but consumed a standardized 500 ml of water immediately upon waking (CON) or 2 hours prior to testing (SDEP). The baseline cognitive assessment commenced at 7.30 for CON and at 6.30 for SDEP, and always began with a psychomotor vigilance task (PVT), followed by the N-back task as described below. Participants were then fitted with all markers, and approximately 30 minutes later, the walking trials commenced.

### 2.3. Cognitive and motor tasks

#### 2.3.1. Psychomotor vigilance task

Sustained attention was assessed using a 10-minute PVT. The PVT is highly sensitive to sleep loss and was included as a baseline measure to verify the effectiveness of the SDEP manipulation [25]. The task was performed at least 30 minutes after waking to minimize sleep inertia [26]. Participants responded as quickly as possible to visual stimuli presented at random intervals on a computer screen. Reaction times and lapses (responses greater than 500 ms) were recorded.

#### 2.3.2. N-back task

Working memory was evaluated using a 2-back task with numeric stimuli (figure 1C) on two occasions: (i) in the morning, seated, as a baseline cognitive assessment, and (ii) as a concurrent cognitive task during walking [27]. Participants were instructed to respond via button press whenever the current number matched the one presented two trials previously. Stimuli were presented at fixed 1.5-second intervals over a 2-minute testing period. Both response accuracy and reaction time were recorded.

#### 2.3.3. Walking task

Participants then completed two 2-minute walking trials on an instrumented split-belt treadmill (M-Gait, Motek, Amsterdam, The Netherlands), interspersed with 1 minute of rest. Treadmill speed was set to each participant's preferred walking speed as determined during the habituation session (mean (s.d.) = 1.25 (0.14) m s^−1^). The trials were performed in a fixed order, with the single-task condition preceding the dual-task condition. During the first trial (single-task), participants were instructed to focus on a point ahead to minimize head movements and to remain in the centre of the treadmill. The second trial included a concurrent 2-back task (dual-task condition, DT), with numeric stimuli presented on a small screen mounted at eye level. Responses were recorded via a wired handheld button. A single handrail was positioned at the front of the treadmill, with no handrails at the sides; participants were instructed not to use the handrail during the trials.

### 2.4. NASA Task Load Index

Upon completion of the testing protocol, participants’ perceived workload was assessed using the NASA Task Load Index (NASA-TLX) questionnaire to evaluate the perceived workload associated with the dual-task walking condition. The questionnaire included the mental demand, physical demand, frustration, performance and effort subscales [28].

### 2.5. Instrumentation and data acquisition

#### 2.5.1. Polysomnography and actigraphy

PSG data were collected using a mobile electroencephalogram (EEG) system (LiveAmp, Brain Products, Gilching, Germany) at a sampling rate of 1000 Hz. EEG electrodes were placed at Fz, Cz, Pz, Oz, C3 and C4 according to the international 10–20 system, with the reference on the right mastoid and a ground electrode on the forehead. Vertical and horizontal eye movements were recorded using electrooculography (EOG) with electrodes above and below the right eye and at both outer canthi. Submental muscle activity was recorded via a chin electromyogram (EMG). EEG and EOG signals were band-pass filtered between 0.1 and 30 Hz, EMG signals between 10 and 200 Hz, and a 50 Hz notch filter was applied to all channels to reduce electrical interference.

Sleep staging was performed using the YASA toolbox in Python [29], incorporating EEG (Cz), EOG and EMG signals to classify wake, N1, N2, N3 and rapid eye movement (REM) stages. Key sleep metrics including total sleep time, sleep efficiency, sleep onset latency, wake after sleep onset (WASO), number of awakenings and stage durations were extracted for analysis.

Seven-day accelerometer data preceding the two experimental sessions were processed using the GGIR package in R (version 3.2-6) [30]. Raw acceleration signals were auto-calibrated, non-wear periods detected and metrics of sleep and physical activity derived.

#### 2.5.2. Marker data collection and preprocessing

Twenty-eight reflective markers were placed on anatomical bony landmarks, and 12 technical cluster markers were placed on each thigh and shank (figure 2). Thirteen infrared cameras (Vicon, Oxford Metrics, Oxford, UK) captured the positions of all reflective markers at a sampling rate of 250 Hz. We used OpenSim 4.5 (OpenSim, Stanford, USA) to first scale the Hamner musculoskeletal model based on the subject's dimensions using a neutral standing trial [31] and to subsequently export marker trajectories of the pelvis and lateral foot markers. The body's centre-of-mass (CoM) position was estimated as the average of the four pelvis markers located on the left and right anterior and posterior superior iliac spines [32]. All marker trajectories were low-pass filtered using a zero-phase second-order Butterworth filter with a 6 Hz cut-off frequency. Filtered positions were then differentiated to compute marker velocity.

**Figure 2.**
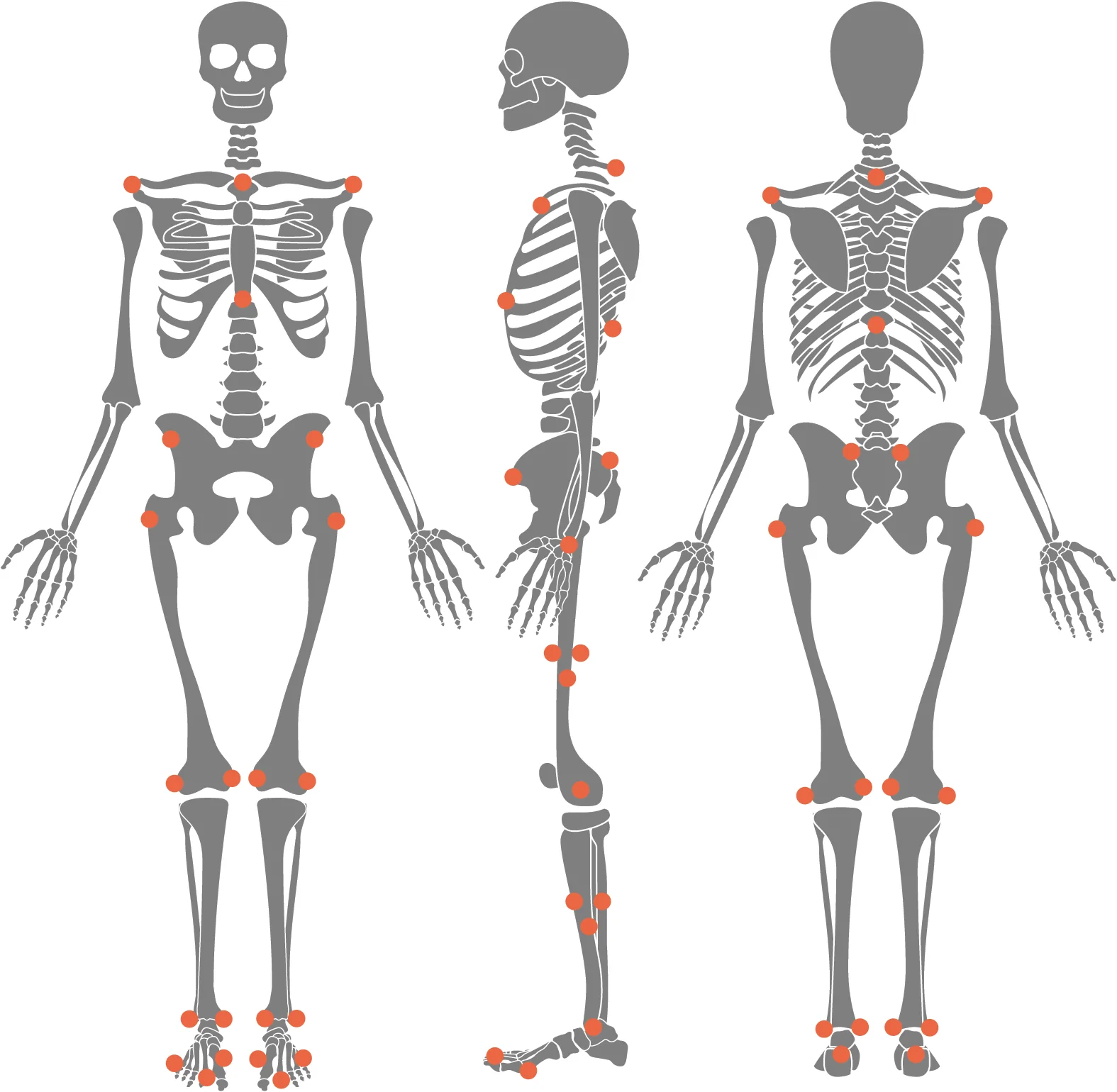
Anatomical location of the reflective markers used for three-dimensional motion capture.

#### 2.5.3. Gait event detection

Heel strikes (HS) and toe-offs (TO) were identified using the AP velocity of the lateral foot markers placed on the fifth metatarsophalangeal joints. HS events were defined as the transition from positive to negative AP velocity, and TO events as the transition from negative to positive velocity [33]. Stance phases were defined as periods between each HS and the subsequent TO. Midstance was defined at 50% of the step. Across all participants, an average (s.d.) of 222.0 (15.72) steps per trial was analysed.

### 2.6. Biomechanical outcome measures

To capture the full spectrum of gait adaptations, we examined three complementary aspects of gait control. First, spatio-temporal parameters (step time, step length and step width) were assessed to characterize overall gait behaviour. Second, foot placement control was examined given its key role in regulating gait stability, with errors quantified separately in the AP and ML directions, as these planes differ in their reliance on active neural regulation. Finally, ML margins of stability were assessed to determine whether any observed changes in spatio-temporal parameters and foot placement control translated into measurable alterations in instantaneous gait stability.

#### 2.6.1. Spatio-temporal parameters

We calculated step time, step length and step width for each step to characterize participants’ general gait patterns. Step time was defined as the temporal interval between successive HS of alternating feet. Step length was calculated using the AP positions of the leading and stance feet at contralateral TO. Similarly, step width was calculated based on the ML distance between lateral foot markers on the leading and stance foot at contralateral TO. We calculated the within-trial mean and standard deviation of step time, step length and step width from their respective time series.

#### 2.6.2. Foot placement control

Based on Wang & Srinivasan [34], a multiple linear regression model was used to assess lateral foot placement control (FP_ML_). The ML position (CoM_pos_ML_) and velocity (CoM_vel_ML_) of the CoM at midstance were included as independent variables, while the ML position of the subsequent contralateral foot (FP_ML_) at HS was included as the dependent variable. Both predictors and outcomes were expressed relative to the ipsilateral stance foot and mean-centred prior to analysis. The degree of foot placement control was quantified as the relative explained variance (*R*^2^) of the linear regression. Foot placement error (FPE) was quantified as the root mean square of the regression residuals, providing a measure of deviation from the predicted foot placement and thus precision of foot placement.

$$\begin{array}{r} {\mathrm{FP}}_{ML}=\beta_{{pos}_{ML}}\cdot {CoM}_{{pos}_{ML}}(i)+\beta_{{vel}_{ML}}\cdot {CoM}_{{vel}_{ML(i)}}+\varepsilon_{ML(i)}. \end{array}$$

Similar to the model for lateral foot placement, a multiple linear regression model for the AP foot placement was fitted. In this case, the AP position of the subsequent contralateral foot (FP_AP_) was predicted based on the AP position (CoM_pos_AP_) and velocity of the CoM (CoM_vel_AP_) at ipsilateral TO.

$$\begin{array}{r} FP_{AP}=\beta_{pos_{AP}}\cdot \;CoM_{pos_{AP\left(i\right)}}+\beta_{vel_{AP}}\cdot \;CoM_{vel_{AP\left(i\right)}}+\varepsilon_{AP\left(i\right)}. \end{array}$$

#### 2.6.3. Mediolateral margins of stability

Dynamic margins of stability (MoS_ML_) were calculated based on the extrapolated centre-of-mass (XCoM) concept [35]. The XCoM was defined as

$$\begin{array}{r} XCoM\left(t\right)=x\left(t\right)+\frac{\dot{x}\left(t\right)}{\omega_{0}}, \end{array}$$

where *x* is the CoM position, $\dot{x}$ is the CoM velocity and *ω*_0_ is the natural frequency of the inverted pendulum

$$\begin{array}{r} \omega_{0}=\surd \left(\frac{g}{l}\right), \end{array}$$

where *g* = 9.81 m s^−2^ represents gravitational acceleration, and *l* is the effective pendulum length, defined as the mean distance from the lateral ankle to the greater trochanter marker during the static trial.

The MoS_ML_ was then defined as the distance between the XCoM and the boundary of the base of support (BoS)

$$\begin{array}{r} MoS_{ML}\left(t\right)=BoS_{ML}-XCoM\left(t\right). \end{array}$$

The boundary of BoS was defined by the ML position of the lateral stance foot marker. Consistent with recommendations, the MoS was extracted at contralateral TO for each step [36] and multiplied by −1 for alternating steps such that positive MoS_ML_ values indicated that the extrapolated CoM remains within the BoS, reflecting mechanically stable steps, whereas negative values indicate that the CoM extends beyond the BoS, reflecting instantaneous mechanical instability. Mean and variability in MoS_ML_ were quantified as the mean and standard deviation from the MoS_ML_ values across all steps within each trial.

### 2.7. Statistics

Linear mixed-effects models (LMMs) evaluated the effects of sleep condition (CON vs SDEP) and task (single- vs dual-task) on all biomechanical outcome measures and N-back task performance. For the N-back analyses, task type reflected whether the assessment was conducted in the morning (seated, no walking) or during the dual-task walking condition. Because the number of correct N-back responses is bounded (0–50), we additionally modelled accuracy using a binomial generalized linear mixed-model (GLMM; successes out of 50 trials; session × trial + order + (1|subject)) as a sensitivity analysis.

Model assumptions were verified for all outcomes: normality of residuals and random effects was assessed using the Shapiro–Wilk test and Q–Q plots, and homoscedasticity was evaluated using a Spearman correlation between absolute residuals and fitted values (see supplementary material). Assumptions were largely satisfied across outcomes; mild violations were observed in variability measures, which is consistent with the typically skewed distribution of such metrics in biomechanical data. Given the balanced repeated-measures design, these mild violations are not expected to meaningfully affect model estimates.

Models included fixed effects for condition and task, their two-way interaction and random effects for subjects. Condition order (CON-first vs SDEP-first) was included as a fixed covariate in all models. It was non-significant for every biomechanical and cognitive outcome (all *p* > 0.05), indicating no evidence of carryover effects. Full order estimates and adjusted *p*-values for all outcomes are reported in supplementary material, table S1. The factor subjects were modelled with a random intercept to account for repeated measures within individuals. To calculate Cohen's *d* pairwise effect sizes, pairwise comparisons between each level of the fixed effects were conducted (see supplementary material, table S2). The comparisons were not used for inferential testing.

To control the familywise error rate, *p*-values were adjusted using the Holm method. Outcomes were grouped into four families based on conceptual similarity: spatio-temporal means (step time, step length, step width), spatio-temporal variability (step time variability, step length variability, step width variability), FPE measures (*R*^2^_ML_, FPE_ML_, *R*^2^_AP_, FPE_AP_) and margin of stability measures (MoS_ML_, MoS_ML_ variability). Correction was applied separately for each term (sleep condition main effect, task main effect and their interaction) within each family.

Lastly, NASA-TLX and PVT scores were analysed using paired-samples *t*-tests. All analyses were performed in R using the lme4 and lmerTest packages. Data are presented as mean (s.d.), and the significance level was set at *p* < 0.05.

## 3. Results

### 3.1. Sleep architecture

PSG recordings during the control night indicated a mean total sleep duration of 7.66 (0.51) hours, with an average sleep efficiency of 84.24 (4.14)%. WASO averaged 42.71 (19.45) minutes. The proportional distribution of sleep stages was: 6.27 (2.98)% for non-rapid eye movement (NREM) stage 1 sleep, 51.19 (5.54)% for stage 2, 19.41 (3.71)% for stage 3, and 23.14 (3.89)% of total sleep time for REM sleep. These values are consistent with normative ranges, indicative of high-quality nocturnal sleep [37].

### 3.2. Cognitive tasks

Median reaction time on the PVT task (figure 3A) was longer following SDEP compared with CON (SDEP: 407 (44.4) ms, CON: 388 (39.2) ms, *t*(17) = –3.02, *p* = 0.008, Cohen's *d* = 0.711). The N-back task was administered both as a seated single-task in the morning and during walking as a dual-task. For the N-back, the task factor included in the LMM contrasted the seated morning assessment with the assessment performed during walking. For the number of correct responses (maximal score = 50), neither sleep condition (*β* = –0.11, 95% CI (–1.35, 1.13), *p* = 0.858) nor task (*β* = 0.17, 95% CI (–1.08, 1.41), *p* = 0.789) had a significant effect, and the sleep condition × task interaction was non-significant (*β* = –0.50, 95% CI (–2.26, 1.26), *p* = 0.571). Median reaction time showed the same pattern. There was no effect of sleep condition (*β* = –6.05 ms, 95% CI (–51.82, 39.71), *p* = 0.792) or task (*β* = 11.93 ms, 95% CI (–33.84, 57.70), *p* = 0.603), and the interaction was non-significant (*β* = 15.27 ms, 95% CI (–49.45, 79.99), *p* = 0.638). Results were unchanged when accuracy was modelled with a binomial GLMM respecting the 0–50 bound (sleep condition *p* = 0.826, task *p* = 0.734, interaction *p* = 0.495). Thus, N-back accuracy and reaction time remained stable across sleep conditions and between the seated and walking assessments.

**Figure 3.**
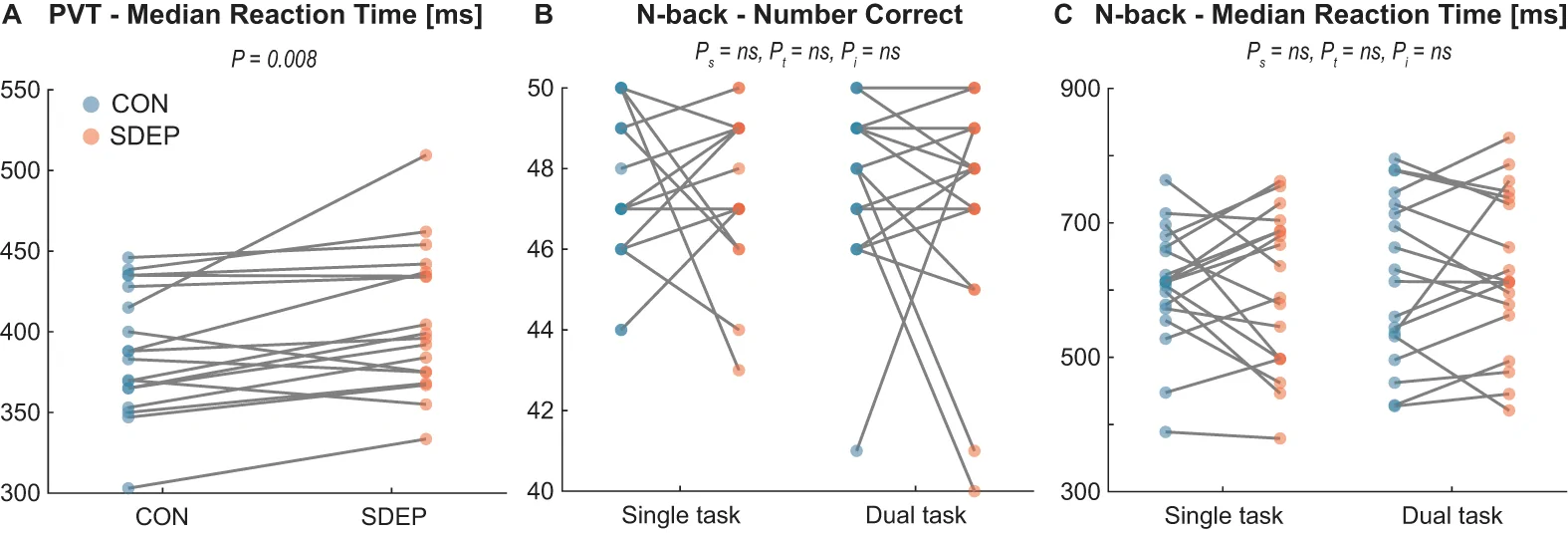
Performance on the cognitive tasks for the control condition (CON) in blue and sleep deprivation condition (SDEP) in red. (A) Median reaction time on the PVT task. (B) Number of correct responses (maximal score = 50) and (C) median reaction time during the N-back task in the single-task condition (seated, in the morning) and dual-task condition (during walking). Each dot represents a participant, with lines linking repeated measurements from the same individual. Condition order was included as a covariate in all models and was non-significant for all outcomes (see supplementary material, table S1). *P_s_* = main effect of sleep condition, *P_t_* = main effect of task, *P_i_* = sleep condition × task interaction effect.

### 3.3. Spatio-temporal parameters

SDEP and DT both influenced spatio-temporal gait parameters, but in distinct ways. SDEP led to a small but significant increase in mean step time (*p* < 0.001, figure 4A) and step length (*p* = 0.002, figure 4B), while step time and step length variability (figure 4D,E), as well as step width and its variability (figure 4C,F), were unaffected. By contrast, performing the N-back task while walking resulted in shorter (*p* = 0.043) and faster steps (*p* = 0.048), along with reduced step time variability (*p* = 0.001) and reduced step width variability (*p* = 0.043), suggesting more consistent stepping under cognitive load. No significant interactions between sleep condition and task were observed for any of the spatio-temporal measures (all *p* > 0.05). Results of the LMM and descriptive statistics can be found in tables 1 and 2.

**Figure 4.**
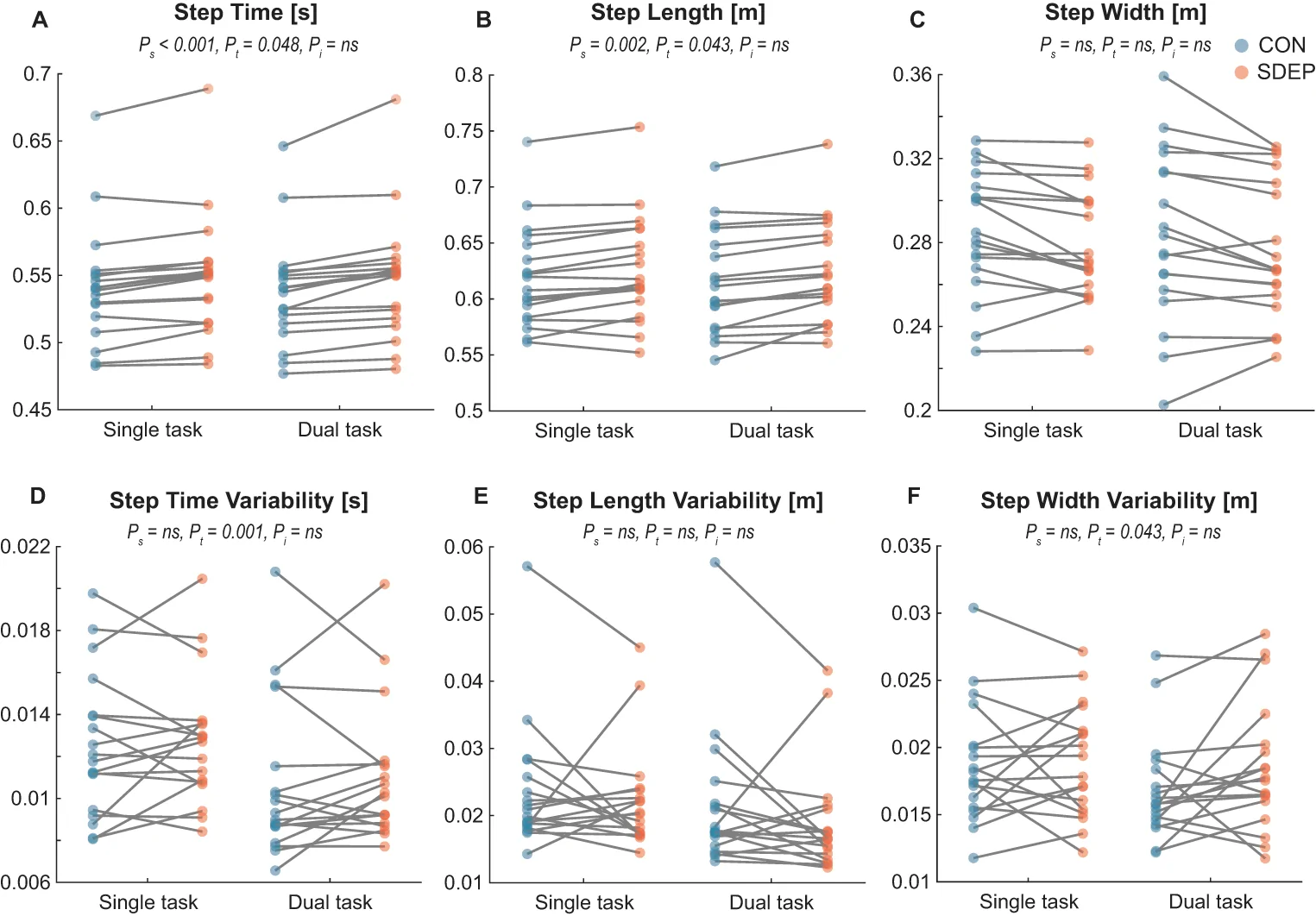
Spatio-temporal walking parameters under control (CON; blue) and sleep deprivation (SDEP; red) conditions. Data are shown for single-task walking (no cognitive task) and dual-task walking (simultaneous N-back performance). Each dot represents an individual participant, with lines connecting repeated measurements from the same participant. Condition order was included as a covariate in all models and was non-significant for all outcomes (see supplementary material, table S1). *P_s_* = main effect of sleep condition, *P_t_* = main effect of task, *P_i_* = sleep condition × task interaction effect.

**Table 1.** LMM results for each outcome, including beta coefficients, standard errors, 95% confidence intervals (CIs) and Holm-adjusted *p*-values.

outcome	term	beta	s.e.	CI	*p*_adj_
step time (s)	sleep condition (ref = control condition)	0.007	0.002	(0.004, 0.011)	**<0.001**
	task (ref = single task)	−0.004	0.002	(−0.008, −0.001)	**0.048**
	sleep condition × task	0.011	0.020	(−0.031, 0.054)	0.682
step time variability (s)	sleep condition (ref = control condition)	0.000	0.000	(−0.001, 0.001)	1.000
	task (ref = single task)	−0.002	0.000	(−0.003, −0.001)	**0.001**
	sleep condition × task	0.001	0.001	(−0.002, 0.004)	1.000
step length (m)	sleep condition (ref = control condition)	0.007	0.002	(0.003, 0.012)	**0.002**
	task (ref = single task)	−0.005	0.002	(−0.009, −0.001)	**0.043**
	sleep condition × task	−0.025	0.020	(−0.068, 0.017)	0.682
step length variability (m)	sleep condition (ref = control condition)	−0.001	0.002	(−0.004, 0.002)	1.000
	task (ref = single task)	−0.002	0.002	(−0.005, 0.001)	0.180
	sleep condition × task	−0.002	0.004	(−0.01, 0.005)	1.000
step width (m)	sleep condition (ref = control condition)	−0.006	0.004	(−0.014, 0.001)	0.107
	task (ref = single task)	−0.002	0.004	(−0.01, 0.006)	0.615
	sleep condition × task	−0.014	0.014	(−0.043, 0.015)	0.682
step width variability (m)	sleep condition (ref = control condition)	0.000	0.001	(−0.002, 0.002)	1.000
	task (ref = single task)	−0.002	0.001	(−0.004, 0)	**0.043**
	sleep condition × task	−0.001	0.002	(−0.005, 0.002)	1.000
*R*^2^_ML_ (%)	sleep condition (ref = control condition)	0.013	0.020	(−0.028, 0.053)	0.727
	task (ref = single task)	0.025	0.020	(−0.016, 0.065)	0.452
	sleep condition × task	0.029	0.049	(−0.075, 0.133)	1.000
FPE_ML_ (m)	sleep condition (ref = control condition)	0.000	0.000	(−0.001, 0)	0.727
	task (ref = single task)	−0.002	0.000	(−0.002, −0.001)	**<0.001**
	sleep condition × task	0.000	0.001	(−0.002, 0.001)	1.000
*R*^2^_AP_ (%)	sleep condition (ref = control condition)	0.052	0.022	(0.007, 0.097)	0.070
	task (ref = single task)	0.009	0.022	(−0.036, 0.053)	0.699
	sleep condition × task	−0.030	0.035	(−0.105, 0.044)	1.000
FPE_AP_ (m)	sleep condition (ref = control condition)	−0.001	0.000	(−0.001, 0)	**0.004**
	task (ref = single task)	−0.002	0.000	(−0.003, −0.002)	**<0.001**
	sleep condition × task	0.000	0.001	(−0.001, 0.001)	1.000
MoS_ML_ (m)	sleep condition (ref = control condition)	−0.004	0.002	(−0.007, −0.001)	**0.025**
	task (ref = single task)	0.000	0.002	(−0.003, 0.003)	0.848
	sleep condition × task	−0.003	0.004	(−0.012, 0.006)	0.504
MoS_ML_ variability (m)	sleep condition (ref = control condition)	−0.001	0.000	(−0.002, 0)	0.090
	task (ref = single task)	−0.002	0.000	(−0.003, −0.001)	**<0.001**
	sleep condition × task	−0.001	0.001	(−0.002, 0.001)	0.504

Note: All results are adjusted for condition order.

**Table 2.** Descriptive statistics (mean (s.d.)) for each outcome by task and sleep condition.

	single-task		dual-task	
outcome	CON	SDEP	CON	SDEP
step time (s)	0.542 (0.044)	0.549 (0.046)	0.538 (0.041)	0.547 (0.046)
step time variability (s)	0.013 (0.003)	0.013 (0.003)	0.011 (0.004)	0.011 (0.003)
step length (m)	0.620 (0.046)	0.627 (0.048)	0.615 (0.046)	0.625 (0.045)
step length variability (m)	0.024 (0.010)	0.022 (0.008)	0.021 (0.010)	0.019 (0.008)
step width (m)	0.285 (0.029)	0.278 (0.026)	0.283 (0.041)	0.276 (0.033)
step width variability (m)	0.019 (0.005)	0.019 (0.004)	0.017 (0.004)	0.019 (0.005)
*R*^2^_ML_ (%)	0.625 (0.130)	0.637 (0.121)	0.649 (0.130)	0.690 (0.104)
FPE_ML_ (m)	0.010 (0.002)	0.010 (0.002)	0.008 (0.001)	0.009 (0.001)
*R*^2^_AP_ (%)	0.429 (0.100)	0.481 (0.113)	0.438 (0.089)	0.460 (0.092)
FPE_AP_ (m)	0.010 (0.002)	0.009 (0.002)	0.008 (0.001)	0.008 (0.002)
MoS_ML_(m)	0.093 (0.093)	0.089 (0.090)	0.010 (0.012)	0.009 (0.011)
MoS_ML_ variability (m)	0.012 (0.010)	0.011 (0.010)	0.002 (0.002)	0.002 (0.002)

### 3.4. Foot placement prediction

SDEP and DT uniquely affected ML and AP foot placement control. For *R*^2^_ML_, neither SDEP (*p* = 0.727) nor DT (*p* = 0.452) had a significant effect, and the interaction was non-significant (figure 5A). However, FPE_ML_ was smaller during DT compared with single-task walking (*p* < 0.001), but SDEP had no effect (*p* = 0.727), and the interaction remained non-significant (figure 5B).

**Figure 5.**
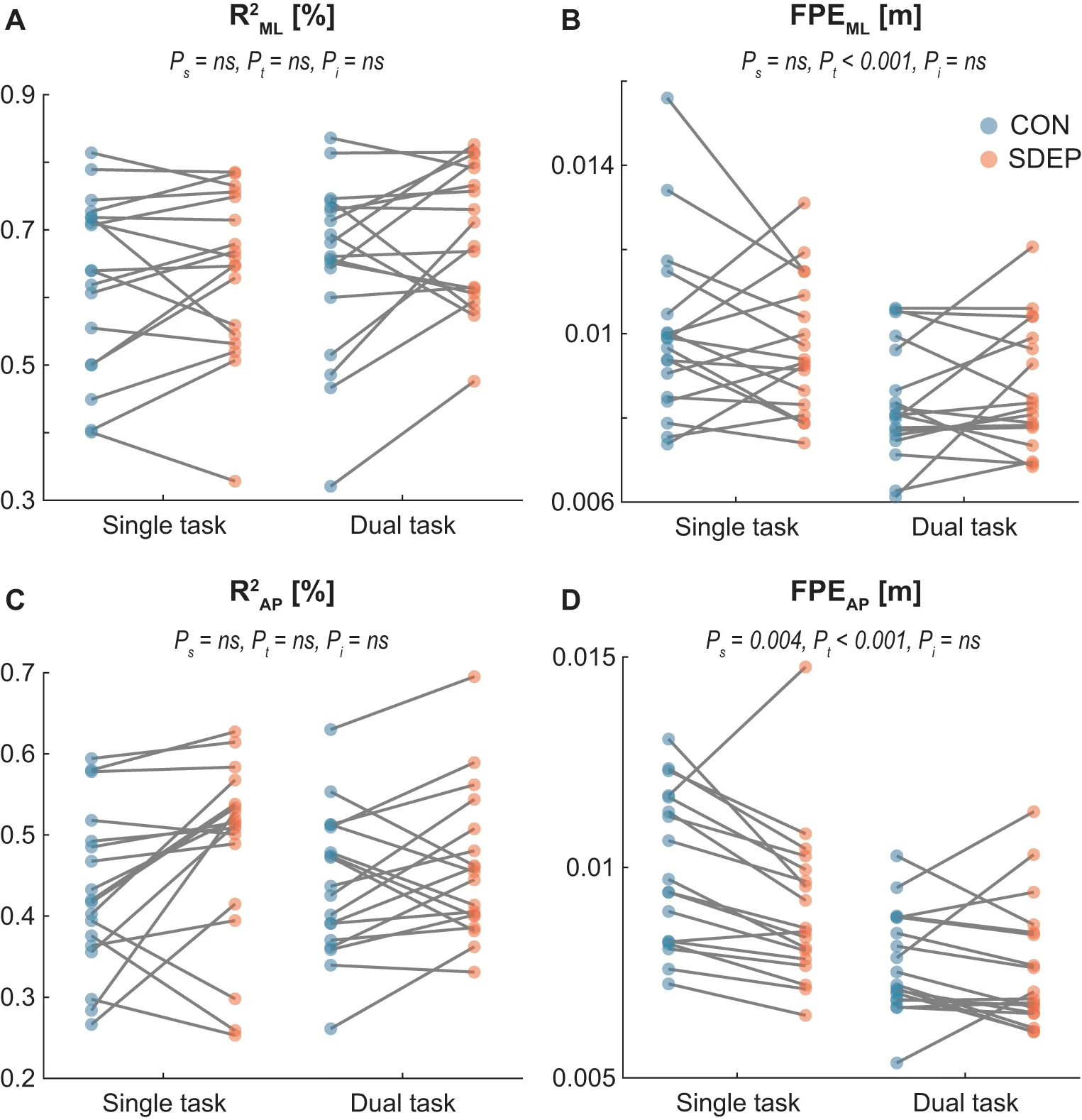
*R*^2^ and foot placement error (FPE) in mediolateral (ML) and anteroposterior (AP) directions under control (CON; blue) and sleep deprivation (SDEP; red) conditions. Data are shown for single-task walking (no cognitive task) and dual-task walking (simultaneous N-back performance). Each dot represents an individual participant, with lines connecting repeated measurements from the same participant. Condition order was included as a covariate in all models and was non-significant for all outcomes (see supplementary material, table S1). *P_s_* = main effect of sleep condition, *P_t_* = main effect of task, *P_i_* = sleep condition × task interaction effect.

In the AP direction, neither SDEP (*p* = 0.070) nor DT (*p* = 0.699) had an effect on *R*^2^, and the interaction was non-significant ( figure 5C). FPE_AP_ was reduced by both SDEP (*p* = 0.004, figure 5D) and DT (*p* < 0.001), and the interaction remained non-significant.

### 3.5. Margins of stability

SDEP led to a decrease in mean MoS_ML_ (*p* = 0.025), indicating slightly reduced ML stability, whereas DT did not significantly affect mean MoS_ML_ (*p* = 0.848, figure 6A). There was no significant interaction between sleep condition and task (*p* = 0.674). In terms of MoS_ML_ variability, DT reduced variability (*p* < 0.001), while SDEP had no significant effect (*p* = 0.090), and the interaction remained non-significant (*p* = 0.346, figure 6B). These results suggest that SDEP subtly decreases stability, whereas cognitive load reduced variability without changing the average MoS_ML_.

**Figure 6.**
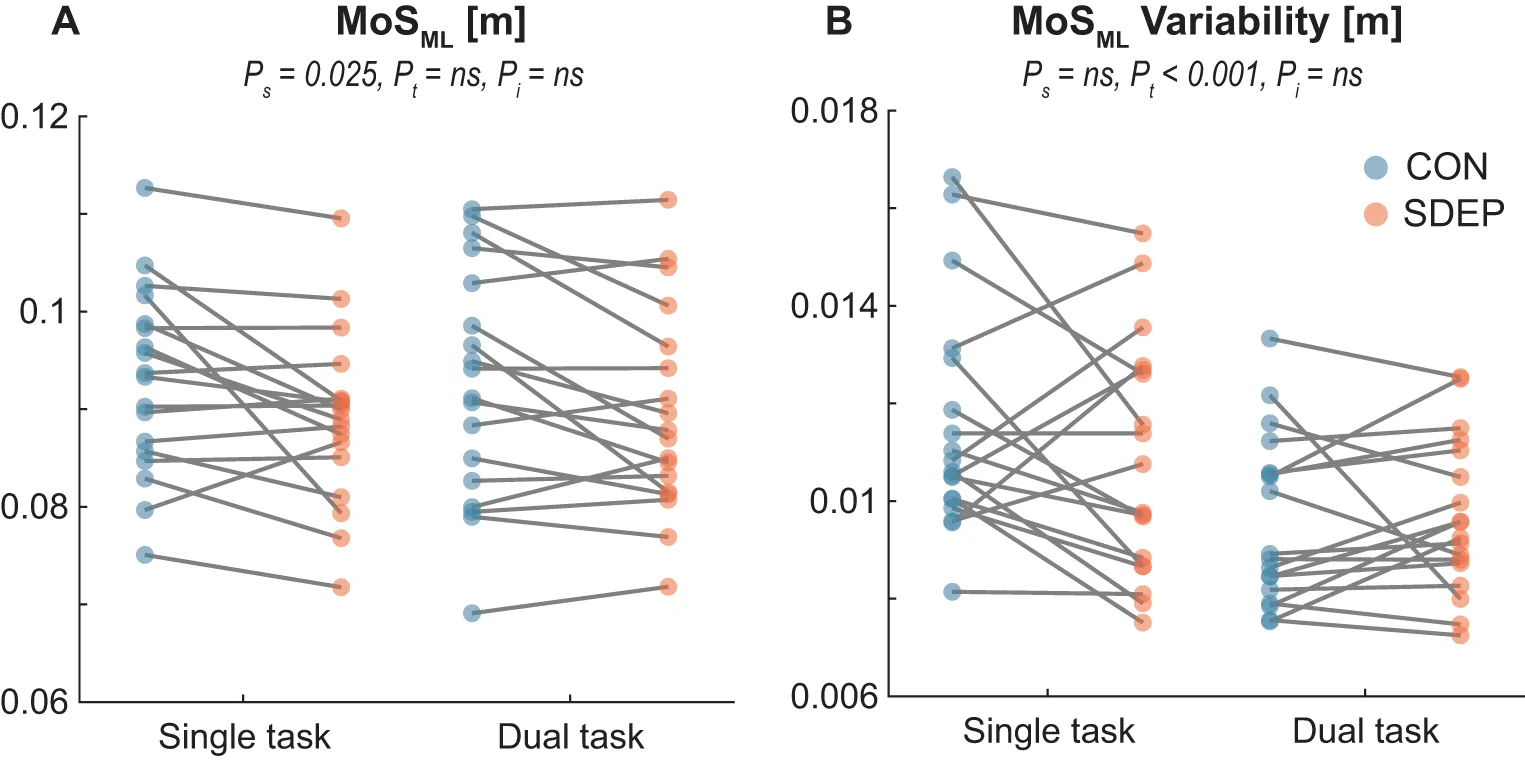
Mean and variability (s.d.) of margin of stability (MoS_ML_) under control (CON; blue) and sleep deprivation (SDEP; red) conditions. Data are shown for single-task walking (no cognitive task) and dual-task walking (simultaneous N-back performance). Each dot represents an individual participant, with lines connecting repeated measurements from the same participant. Condition order was included as a covariate in all models and was non-significant for all outcomes (see supplementary material, table S1). *P_s_* = main effect of sleep condition, *P_t_* = main effect of task, *P_i_* = sleep condition × task interaction effect.

### 3.6. NASA Task Load Index

Subjective workload was assessed using the NASA-TLX immediately after the dual-task walking condition ( table 3). Overall workload, as indicated by the total NASA-TLX score, was higher during SDEP compared with the control condition (*p* < 0.001). Among the subscales, participants reported greater effort (*p* = 0.001) and frustration (*p* = 0.013) during SDEP. Mental demand (*p* = 0.083) and physical demand (*p* = 0.095) showed trends towards higher ratings in SDEP, although these did not reach statistical significance. Performance ratings did not differ significantly between conditions (*p* = 0.539).

**Table 3.** Results of paired-samples *t*-test and descriptive statistics (mean (s.d.)) of the NASA-TLX by sleep condition.

**outcome**	**CON**	**SDEP**	**diff**	***t***	***p*-value**	**Cohen's *d***
mental demand	8.167 (4.902)	10.000 (5.831)	1.833	−1.838	0.084	0.335
physical demand	1.889 (1.676)	3.500 (4.260)	1.611	−1.767	0.095	0.449
performance	3.556 (2.975)	4.111 (2.867)	0.556	−0.627	0.539	0.190
effort	7.778 (4.977)	11.500 (4.878)	3.722	−3.908	0.001	0.755
frustration	4.389 (4.539)	7.333 (5.347)	2.944	−2.773	0.013	0.588
total NASA-TLX	25.778 (13.371)	36.444 (15.912)	10.667	−4.035	0.001	0.712

*Note:* diff, absolute difference between values of control condition (CON) and sleep deprivation condition (SDEP).

## 4. Discussion

The present study investigated the effects of one night of total SDEP on gait control and stability under single- and dual-task walking conditions. We hypothesized that SDEP would produce effects similar to dual-task walking, reflecting a shared underlying mechanism of reduced cognitive resource availability. In addition, we expected the impact of SDEP to be larger in the dual-tasking condition as observed by a significant interaction effect. However, comparing the effects of SDEP and dual-tasking showed mixed results. To obtain a comprehensive view of gait adaptations, we examined gait using three complementary analytical approaches. At the spatio-temporal level, SDEP increased step time, whereas dual-tasking decreased it. In addition, dual-tasking primarily reduced gait variability, whereas SDEP did not affect variability. These measures describe overall changes in gait behaviour but do not directly reflect underlying control strategies. Therefore, we next examined foot placement control, given its key role in stabilizing gait [34]. We found decreased FPE_AP_ under both dual-tasking and SDEP, whereas FPE_ML_ was only affected by dual-tasking. Finally, to determine whether these changes translated into altered balance control, we assessed MoS_ML_. MoS_ML_ was reduced following SDEP but remained unaltered during dual-tasking, suggesting that despite changes in spatio-temporal parameters and foot placement control in both conditions, only SDEP resulted in measurable impairments in ML instantaneous stability. Overall, the present results suggest the effects of dual-tasking and SDEP on gait arise from distinct underlying mechanisms. More specifically, SDEP might (i) impact different subcomponents of cognitive functions compared with dual-tasking and/or (ii) affect gait through mechanisms that extend beyond cognitive factors.

### 4.1. Subcomponents of attention

Although both SDEP and dual-tasking impact attention, they might do so by affecting different subcomponents of attention, which may explain the divergent gait adaptations observed. The differential effects of SDEP and dual-tasking can be understood using van Zomeren and Brouwer's multi-component model of attention, which distinguishes intensity and selectivity dimensions [38]. SDEP primarily compromises the *intensity* components, that is, tonic alertness and sustained vigilance, reducing overall arousal and producing a global reduction in the system's capacity. This effect manifests as pronounced impairments in basic, continuous processing tasks, while higher order functions such as working memory and reasoning are relatively spared [39–42]. Consistent with this, participants in the present study showed increased median reaction times on the PVT following total SDEP (figure 3A), whereas performance on the 2-back task remained stable (figure 3B). By contrast, dual-tasking challenges the *selectivity* dimension by requiring the flexible allocation and coordination of attentional resources across concurrent activities via executive control. Performance decrements under dual-tasking therefore arise from competition for shared cognitive resources within an otherwise alert system. Since vigilance and sustained attention are also required for continuous gait monitoring and control, the intensity deficit induced by SDEP could plausibly drive the observed gait adaptations through a distinct cognitive pathway from that challenged by dual-tasking. However, it is important to note that the stability of 2-back performance under SDEP also means we cannot fully rule out that the gait changes following SDEP are driven by non-cognitive mechanisms, such as altered sensorimotor processing, or changes in neuromuscular control. Distinguishing between these cognitive and non-cognitive explanations remains an important direction for future research.

### 4.2. Gait adaptations under dual-tasking

Given that attention and executive functions are necessary for stable gait, impairments in both types of attention may pose a threat to stability and negatively impact gait [43]. When dynamic stability during gait is challenged (e.g. by perturbations or uneven terrain), individuals typically adopt a protective gait pattern characterized by shorter and wider steps [44,45]. Increasing step width enhances the MoS_ML_, while shorter steps help maintain the CoM within this margin and reduce vulnerability to slips or trips [46]. Thus, shorter and wider steps are generally interpreted as a stability-enhancing strategy. The strategy of increasing step width has also been reported in clinical populations, which has been interpreted as a compensatory response to counteract the impaired postural control associated with these disorders [47,48].

This protective pattern was somehow evident in the dual-task condition in the current study. Apart from taking shorter steps (figure 4A), sleep-deprived participants also reduced their spatio-temporal variability (figure 4D,F). Gait variability reflects the extent to which the motor system explores different movement solutions across steps. Reduced spatio-temporal variability indicates tighter neuromotor regulation, often described as a stiffer control strategy, which limits adaptability but reduces unexpected deviations that could compromise balance. This stiffer control strategy is also reflected in the lower FPE in both the AP and ML directions (figure 5B,D). FPE quantifies how closely actual foot placement matches the placement predicted to regulate the CoM. Lower FPE indicates tighter coupling between foot placement and the instantaneous CoM state, reflecting reduced tolerance for deviation and increased reliance on predictive, tightly regulated control rather than adaptive or exploratory behaviour. Taken together, these adaptations could be interpreted as a stiffer, less adaptable, yet seemingly ‘safer’ walking pattern.

Alternatively, the gait adaptation during dual-tasking may reflect a shift towards a more automatic control strategy in response to the reduced attentional selectivity. Because dual-tasking taxes executive functions responsible for task coordination and flexible resource allocation, actively controlling gait becomes more costly. To mitigate this executive load, the motor system may adopt a simplified and more stereotyped gait pattern that relies more heavily on automatic, subcortical control processes and less on continuous executive supervision. Doing so, cognitive resources can be reallocated towards the secondary cognitive task. This interpretation is supported by postural control studies in young adults showing reduced sway while standing when a dual-task was added [49–52]. Importantly, these reductions occurred without increases in ankle muscle activity, indicating that stabilization is not achieved through stiffening but rather through greater reliance on automatic control processes [53]. These studies therefore suggest that the shift from an internal to an external focus during dual-tasking can enhance the automaticity and stability of postural control. Thus, in this population, dual-tasking may not necessarily pose a threat to gait stability or lead to a protective gait pattern. Instead, dual-tasking may be associated with a shift towards a more automatic gait pattern that helps preserve stability.

### 4.3. Gait adaptations under sleep deprivation

By contrast to dual-tasking, participants in the SDEP condition exhibited reduced instantaneous gait stability, reflected by a lower average MoS_ML_ (figure 6A). Sleep-deprived participants also adopted longer steps and a slower cadence (figure 4A,B). At first glance, longer steps might appear to represent a stability-enhancing adaptation because they increase the AP base of support. However, ML stability is governed primarily by ML foot placement and the regulation of ML CoM dynamics rather than by AP foot placement [21–23]. Consequently, increases in step length do not necessarily translate into improved ML stability. Indeed, the gait pattern observed here differs from the shorter, wider steps that are typically adopted when ML stability is challenged [44,45]. Rather, these adaptations may reflect an effort to conserve energy and walk as passively as possible. Walking with shorter and wider steps is metabolically costly, indicating that optimizing stability carries an energetic penalty [54–56]. In line with this, the observed decrease in FPE_AP_ (figure 5D) following SDEP could reflect a strategy to limit push-off torque around the ankle joint, as FPEs in the AP direction are compensated by increasing push-off torque in the ankle [57]. Therefore, sleep-deprived participants might try to limit their errors in an attempt to avoid the increased torques around the ankle joint. Together, these gait adaptations came at the cost of instantaneous gait stability.

Despite the reduced instantaneous lateral stability, participants did not exhibit active adjustments in FPE_ML_ or step width (figures 4C and 5B) that might have helped preserve stability. MoS_ML_ is determined by both CoM dynamics and the BoS boundary. Since step width, which defines the base of support, remained unchanged, the reduction in MoS_ML_ is unlikely owing to altered foot placement and instead points to changes in CoM dynamics. This result suggests that, under SDEP, participants may prioritize maintaining consistent step width, which is associated with energetic demands during walking, while exhibiting subtle deficits in the regulation of ML CoM motion [58].

An additional consideration is the experimental context of treadmill walking. Previous work has shown that individuals exhibit substantial fluctuations in AP position while walking on a treadmill, suggesting that maintaining a fixed AP position is not tightly regulated during treadmill locomotion [59]. By contrast, the split-belt treadmill may have imposed greater constraints on ML foot placement, as participants were asked to keep each foot on its respective belt, avoid stepping into the gap between belts, and remain centred on the treadmill. Consistent with this interpretation, foot placement control has been shown to decrease during split-belt compared with single-belt treadmill walking [60]. Similarly, imposing spatial constraints through prescribed walking paths on a treadmill has been shown to lower foot placement control [61]. Together, these findings suggest that the task-specific constraints of split-belt treadmill walking may have limited the expression of ML foot placement adaptations, potentially explaining why SDEP affected AP foot placement control while ML foot placement measures remained unchanged.

The observed adaptation in gait may arise from impaired sensorimotor control processes. SDEP is known to reduce alertness, which may compromise feedback control by delaying or weakening the detection and correction of gait errors. Reduced alertness may also impair feed-forward mechanisms by limiting the ability to update and adapt predictive motor plans based on recent sensory information. This interpretation is in line with research showing that feedback and feed-forward processes of motor control are indeed impacted by sleep loss. In the study by Umemura *et al.* [18], participants walked on a treadmill while synchronizing their footsteps with auditory cues after one night of SDEP. They observed a significant increase in period errors, that is, the difference in duration between two consecutive steps and two consecutive auditory cues, which the authors link to deficits in feed-forward control [62]. In another study, decreased performance on a visually guided force-control task was found after sleep restriction [63]. As this task depends on sustained attention and feedback-based corrections, its impairment provides additional evidence that feedback-related sensorimotor processes are disrupted by sleep loss. Thus, the attentional intensity deficit induced by SDEP may impair the ability to generate active feedback- and feed-forward-based corrections, especially in the ML plane. Effective corrections in the ML plane require a heightened level of control and vigilance compared with the AP plane, which may explain why FPE was adjusted in the AP plane but remained unchanged in the ML plane [21–23]. This lack of active corrections and passive gait dynamics under SDEP might make subjects more susceptible to perturbations. Future studies should incorporate external perturbations to directly test this hypothesis.

### 4.4. Limitations

Several limitations should be considered when interpreting the results of this study. First, although each trial included a sufficient number of steps for reliable gait analysis, the relatively short trial duration may have limited the expression of SDEP-related gait alterations. Participants may have been able to temporarily compensate and maintain performance over the brief testing period, whereas longer or repeated trials might reveal effects associated with accelerated mental or physical fatigue induced by SDEP.

Second, the absence of changes in cognitive task performance suggests that the choice of cognitive task may not have been optimal for detecting the effects of SDEP. SDEP has been shown to disproportionately impair sustained attention compared with certain aspects of executive control [39–42], and the task used in this study may not have sufficiently taxed this sustained attention. Alternatively, some studies suggest that individuals undergoing SDEP may allocate more compensatory effort to easier tasks and choose to detach from more challenging tasks [64]. Therefore, the task difficulty in this study may have been too low, allowing participants to maintain performance through compensatory strategies.

Third, the fixed order of the single-task and dual-task conditions represents an important limitation when comparing performance across tasks. Because task order was not counterbalanced, potential order effects cannot be fully disentangled from true task-related differences. Two considerations partially mitigate this concern: (i) participants completed a dedicated habituation session before testing, and (ii) both trials consisted of only 2 minutes of walking at preferred speed, making meaningful physical fatigue in our population unlikely. Nevertheless, a settling-in effect cannot be fully excluded, and could contribute to more consistent, automatic walking in the second trial. This pattern partially resembles several of our dual-task findings, including the decrease in spatio-temporal variability. Future studies should therefore counterbalance task order to directly address this confound.

Fourth, it is important to note that correlations between the CoM state and foot placement alone cannot determine whether the observed relationship reflects active control mechanisms or passive biomechanical dynamics [65]. While such correlations provide insight into gait patterns, they do not by themselves reveal whether adjustments are the result of deliberate, feedback-driven control or emergent passive stability strategies.

Fifth, the relatively small sample size (*n* = 18) limits statistical power, particularly for detecting small interaction effects. Consequently, the absence of significant interactions should therefore be interpreted with caution. Effect sizes are reported throughout to aid interpretation. In addition, all participants were young healthy adults, which limits generalizability to older adults who may show more pronounced gait alterations.

Sixth, the experiment was conducted in a highly controlled laboratory setting, which may limit ecological validity. Participants slept in the laboratory during the control condition, which could have reduced sleep quality compared with a typical night at home. In addition, walking on a treadmill at a fixed speed removes natural self-pacing as an adaptation strategy and may impose a mild attentional demand even in the single-task condition, potentially underestimating effects that might be observed during overground walking.

Finally, while acute SDEP provides a controlled experimental model, it may not fully represent the chronic, partial sleep restriction commonly experienced in real-world settings. Importantly, chronic sleep restriction is associated with a progressive loss of insight into one's own impairment and slower recovery compared with acute deprivation [66–69], suggesting it may represent a more insidious and persistent risk. Whether similar gait alterations emerge under chronic partial sleep restriction remains an important direction for future research.

## 5. Conclusions

In summary, the present study demonstrates that one night of total SDEP and dual-tasking have distinct effects on gait control and stability. Dual-tasking led to a reduction in step time, step length, spatio-temporal variability and FPE in both ML and AP planes, while preserving instantaneous gait stability. By contrast, SDEP resulted in longer steps, increased reliance on the CoM state in the AP plane and decreased instantaneous gait stability. These results suggest that gait adaptations following sleep loss cannot be fully explained by reduced cognitive resource availability.

## Supplementary Material



## Data Availability

The dataset supporting this study contains pseudonymized human participant data and cannot be made fully open access for ethical and legal reasons, in accordance with the informed consent approved by the institutional ethics committee and GDPR requirements. The data are registered in KU Leuven's Research Data Repository (RDR) at [70] and are available on request by submitting an access request to the repository. Access is granted upon approval by KU Leuven, including review by the Data Protection Officer and completion of a Data Transfer Agreement. Supplementary material is available online [71].
