# Supplementary material for: Effects of one night of sleep deprivation on single- and dual-task gait
Source: J R Soc Interface. 2026 Oct 7;23(243):20260047. doi: 10.1098/rsif.2026.0047 (PMC13641949; doi:10.1098/rsif.2026.0047)
Supplement: Supplementary file 1 [file rsif.2026.0047.zip › rsif20260047_si_001.docx]

**Supplementary Material**

**Title:** Effects of one night of sleep deprivation on single- and dual-task gait

**Authors:** Maura Seynaeve, Anna C Render, Peter C Fino, Toon de Beukelaar

**Journal:** Journal of the Royal Society Interface

**Article DOI:** 10.1098/rsif.2026.0047

**Table S1.** Linear mixed-effects model results for each outcome, including condition order as a covariate, with β coefficients, standard errors, 95% confidence intervals (CIs), and Holm-adjusted p-values.

| Outcome | Term | Beta | SE | CI | Padj |
| --- | --- | --- | --- | --- | --- |
| Step time (s) | Sleep condition (ref = Control condition) | 0.007 | 0.002 | [0.004, 0.011] | **<0.001** |
|  | Task (ref = Single Task) | -0.004 | 0.002 | [-0.008, -0.001] | **0.048** |
|  | Sleep condition x Task | 0.011 | 0.020 | [-0.031, 0.054] | 0.682 |
|  | Condition order | 0.002 | 0.003 | [-0.003, 0.007] | 1.000 |
| Step time variability (s) | Sleep condition (ref = Control condition) | 0.000 | 0.000 | [-0.001, 0.001] | 1.000 |
|  | Task (ref = Single Task) | -0.002 | 0.000 | [-0.003, -0.001] | **0.001** |
|  | Sleep condition x Task | 0.001 | 0.001 | [-0.002, 0.004] | 1.000 |
|  | Condition order | 0.000 | 0.001 | [-0.001, 0.001] | 1.000 |
| Step length (m) | Sleep condition (ref = Control condition) | 0.007 | 0.002 | [0.003, 0.012] | **0.002** |
|  | Task (ref = Single Task) | -0.005 | 0.002 | [-0.009, -0.001] | **0.043** |
|  | Sleep condition x Task | -0.025 | 0.020 | [-0.068, 0.017] | 0.682 |
|  | Condition order | 0.003 | 0.003 | [-0.003, 0.008] | 1.000 |
| Step length variability (m) | Sleep condition (ref = Control condition) | -0.001 | 0.002 | [-0.004, 0.002] | 1.000 |
|  | Task (ref = Single Task) | -0.002 | 0.002 | [-0.005, 0.001] | 0.180 |
|  | Sleep condition x Task | -0.002 | 0.004 | [-0.01, 0.005] | 1.000 |
|  | Condition order | -0.001 | 0.002 | [-0.006, 0.003] | 1.000 |
| Step width (m) | Sleep condition (ref = Control condition) | -0.006 | 0.004 | [-0.014, 0.001] | 0.107 |
|  | Task (ref = Single Task) | -0.002 | 0.004 | [-0.01, 0.006] | 0.615 |
|  | Sleep condition x Task | -0.014 | 0.014 | [-0.043, 0.015] | 0.682 |
|  | Condition order | 0.000 | 0.005 | [-0.011, 0.011] | 1.000 |
| Step width variability (m) | Sleep condition (ref = Control condition) | 0.000 | 0.001 | [-0.002, 0.002] | 1.000 |
|  | Task (ref = Single Task) | -0.002 | 0.001 | [-0.004, 0] | **0.043** |
|  | Sleep condition x Task | -0.001 | 0.002 | [-0.005, 0.002] | 1.000 |
|  | Condition order | 0.002 | 0.001 | [-0.001, 0.004] | 0.537 |
| R^2^_ML_ (%) | Sleep condition (ref = Control condition) | 0.013 | 0.020 | [-0.028, 0.053] | 0.727 |
|  | Task (ref = Single Task) | 0.025 | 0.020 | [-0.016, 0.065] | 0.452 |
|  | Sleep condition x Task | 0.029 | 0.049 | [-0.075, 0.133] | 1.000 |
|  | Condition order | 0.028 | 0.029 | [-0.03, 0.085] | 0.677 |
| FPE_ML_ (m) | Sleep condition (ref = Control condition) | 0.000 | 0.000 | [-0.001, 0] | 0.727 |
|  | Task (ref = Single Task) | -0.002 | 0.000 | [-0.002, -0.001] | **<0.001** |
|  | Sleep condition x Task | 0.000 | 0.001 | [-0.002, 0.001] | 1.000 |
|  | Condition order | 0.001 | 0.000 | [0, 0.002] | 0.498 |
| R^2^_AP_ (%) | Sleep condition (ref = Control condition) | 0.052 | 0.022 | [0.007, 0.097] | 0.070 |
|  | Task (ref = Single Task) | 0.009 | 0.022 | [-0.036, 0.053] | 0.699 |
|  | Sleep condition x Task | -0.030 | 0.035 | [-0.105, 0.044] | 1.000 |
|  | Condition order | -0.030 | 0.031 | [-0.093, 0.033] | 0.677 |
| FPE_AP_ (m) | Sleep condition (ref = Control condition) | -0.001 | 0.000 | [-0.001, 0] | **0.004** |
|  | Task (ref = Single Task) | -0.002 | 0.000 | [-0.003, -0.002] | **<0.001** |
|  | Sleep condition x Task | 0.000 | 0.001 | [-0.001, 0.001] | 1.000 |
|  | Condition order | 0.001 | 0.000 | [0, 0.002] | 0.119 |
| MoS_ML_ (m) | Sleep condition (ref = Control condition) | -0.004 | 0.002 | [-0.007, -0.001] | **0.025** |
|  | Task (ref = Single Task) | 0.000 | 0.002 | [-0.003, 0.003] | 0.848 |
|  | Sleep condition x Task | -0.003 | 0.004 | [-0.012, 0.006] | 0.504 |
|  | Condition order | 0.001 | 0.002 | [-0.003, 0.005] | 0.674 |
| MoS_ML_ variability (m) | Sleep condition (ref = Control condition) | -0.001 | 0.000 | [-0.002, 0] | 0.090 |
|  | Task (ref = Single Task) | -0.002 | 0.000 | [-0.003, -0.001] | **<0.001** |
|  | Sleep condition x Task | -0.001 | 0.001 | [-0.002, 0.001] | 0.504 |
|  | Condition order | 0.001 | 0.001 | [0, 0.002] | 0.346 |
