# Supplementary material for: Effects of one night of sleep deprivation on single- and dual-task gait
Source: J R Soc Interface. 2026 Oct 7;23(243):20260047. doi: 10.1098/rsif.2026.0047 (PMC13641949; doi:10.1098/rsif.2026.0047)
Supplement: Supplementary file 1 [file rsif.2026.0047.zip › rsif20260047_si_003.pdf]

Supplementary Material

Title: Effects of one night of sleep deprivation on single- and dual-task gait

Authors: Maura Seynaeve, Anna C Render, Peter C Fino, Toon de Beukelaar

Journal: Journal of the Royal Society Interface

Article DOI: 10.1098/rsif.2026.0047

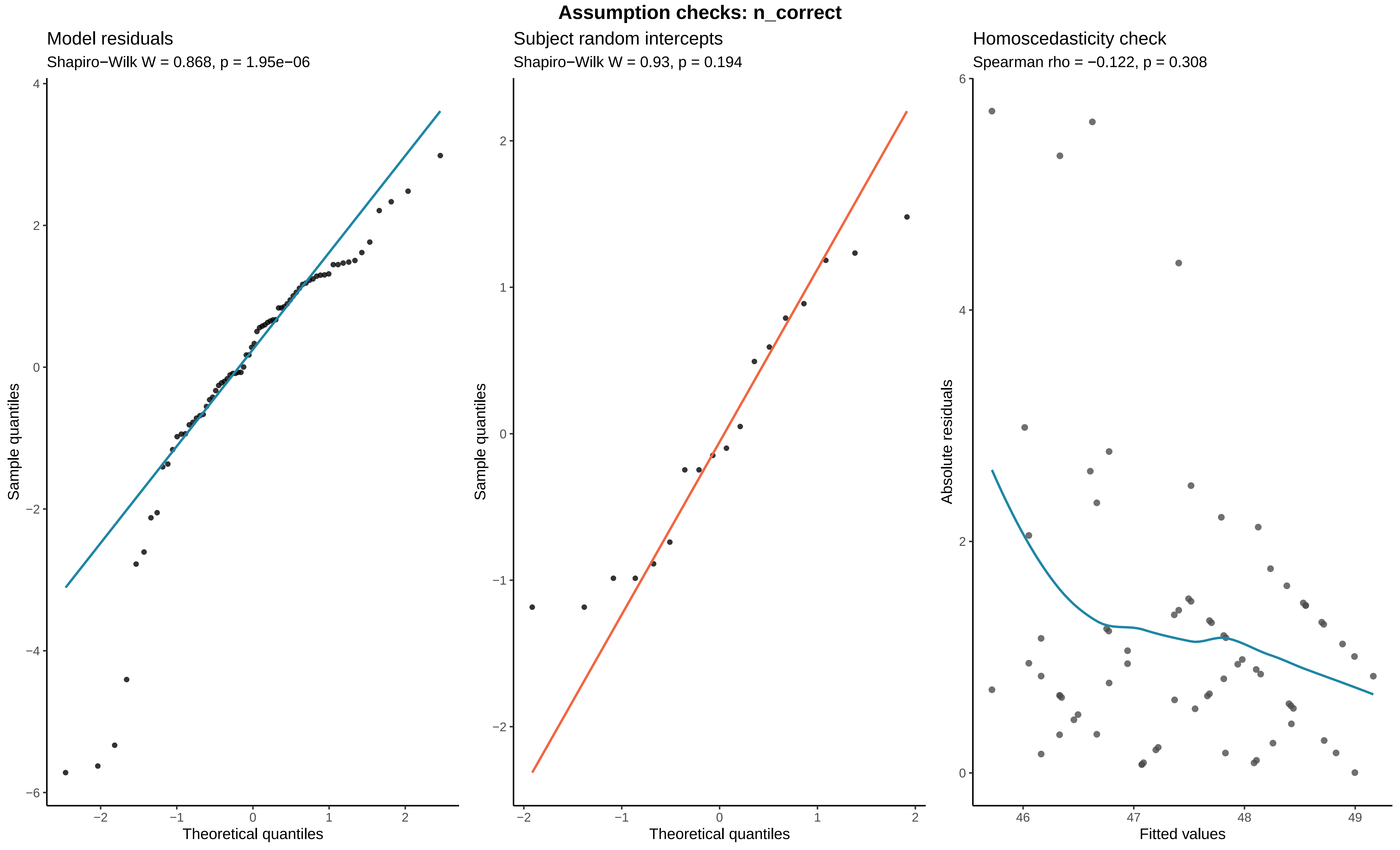
Assumption checks: n_correct
Model residuals
Shapiro−Wilk W = 0.868, p = 1.95e−06
Sample quantiles
Theoretical quantiles
Subject random intercepts
Shapiro−Wilk W = 0.93, p = 0.194
Sample quantiles
Theoretical quantiles
Homoscedasticity check
Spearman rho = −0.122, p = 0.308
Absolute residuals
Fitted values

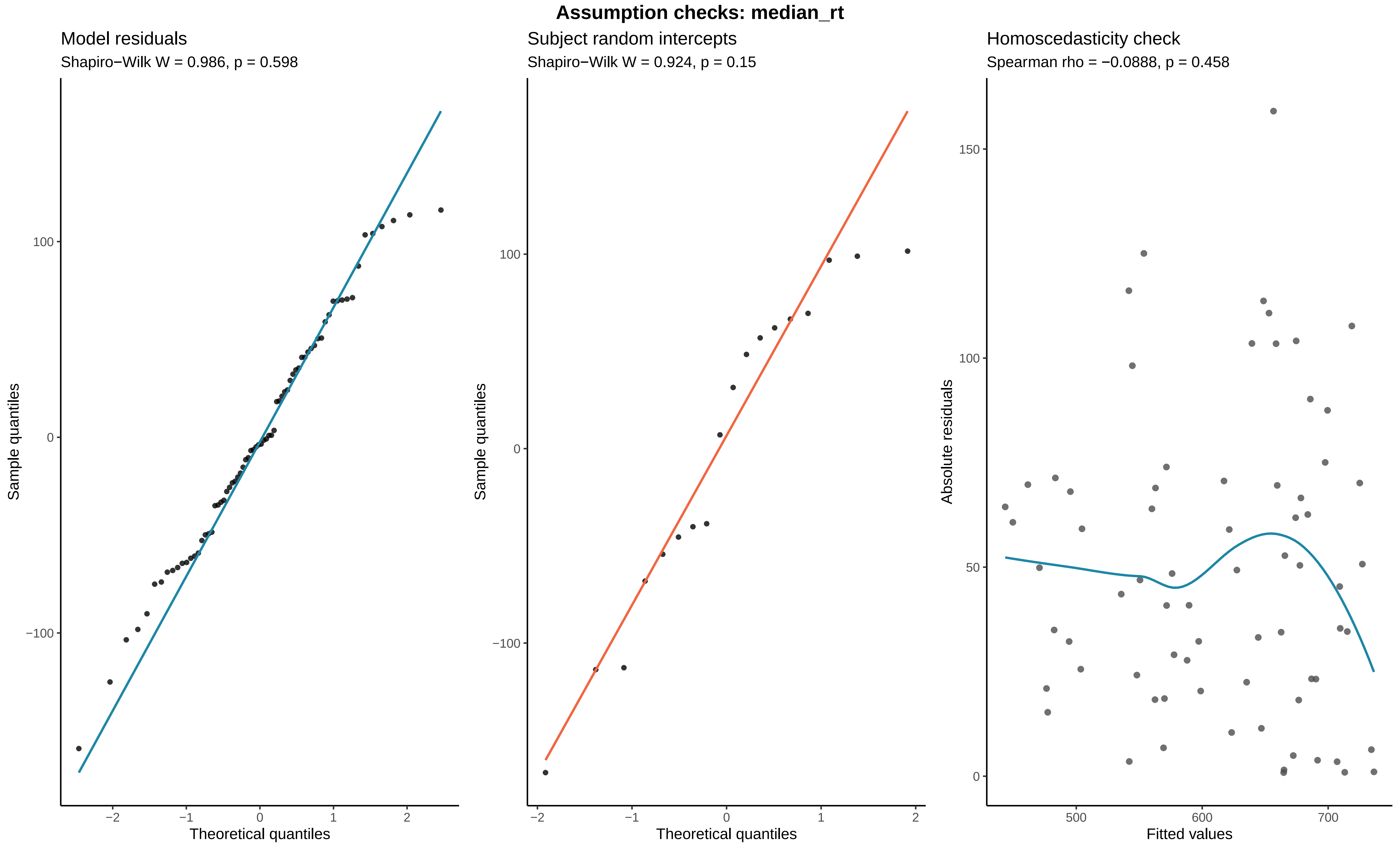

Assumption checks: median_rt
Model residuals
Shapiro−Wilk W = 0.986, p = 0.598
Sample quantiles
Theoretical quantiles
Subject random intercepts
Shapiro−Wilk W = 0.924, p = 0.15
Sample quantiles
Theoretical quantiles
Homoscedasticity check
Spearman rho = −0.0888, p = 0.458
Absolute residuals
Fitted values

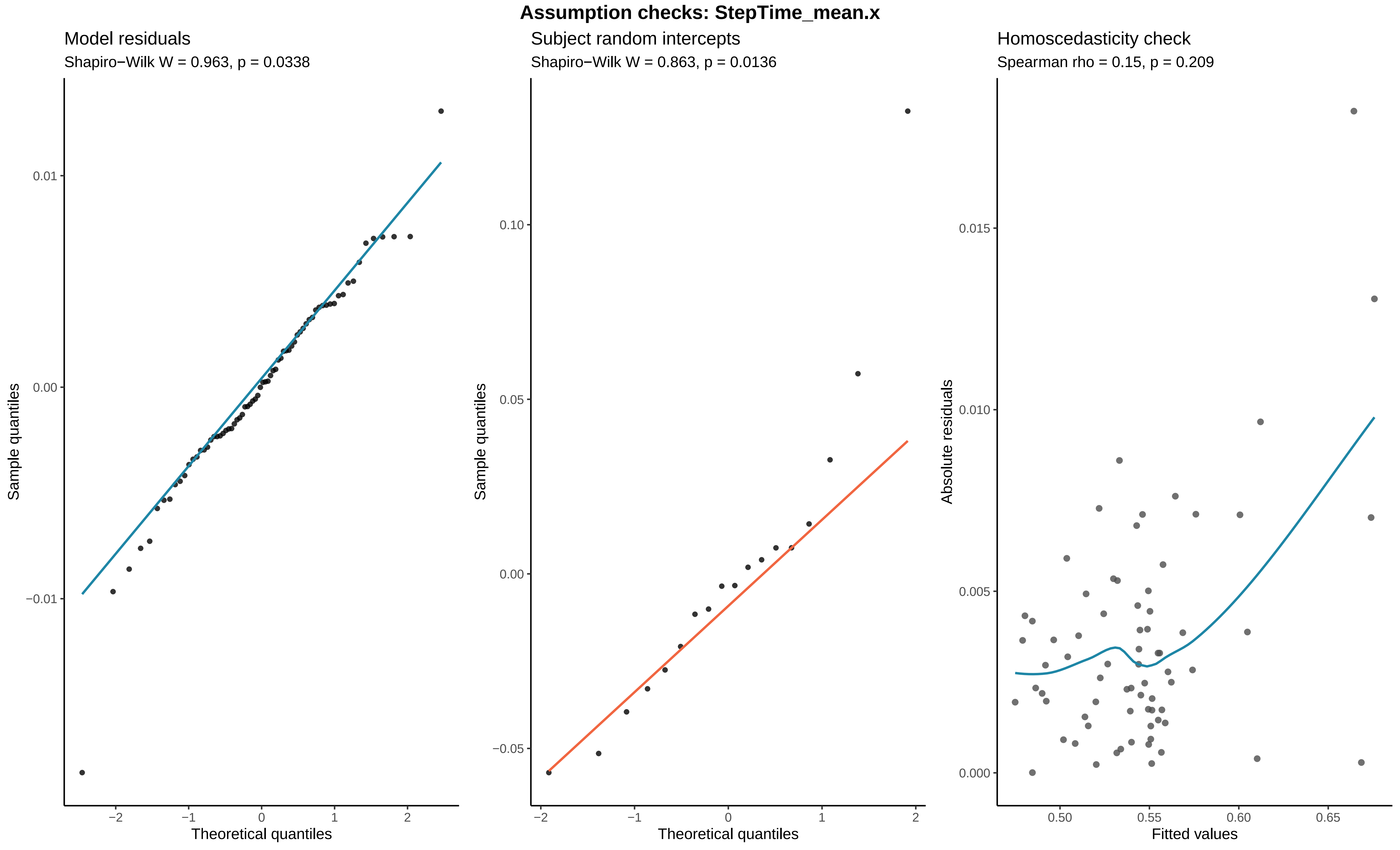
Assumption checks: StepTime_mean.x
Model residuals
Shapiro−Wilk W = 0.963, p = 0.0338
Sample quantiles
Theoretical quantiles
Subject random intercepts
Shapiro−Wilk W = 0.863, p = 0.0136
Sample quantiles
Theoretical quantiles
Homoscedasticity check
Spearman rho = 0.15, p = 0.209
Absolute residuals
Fitted values

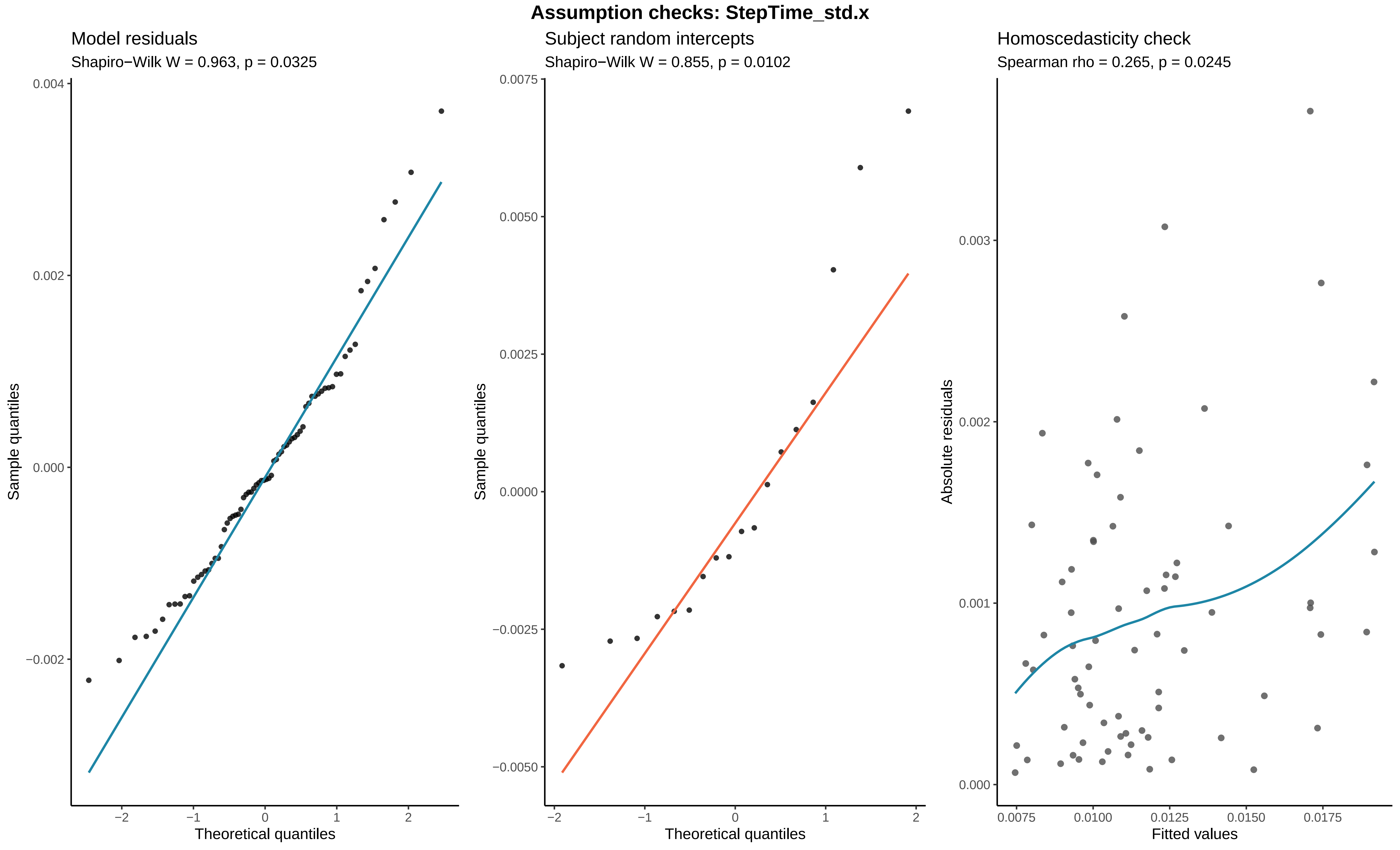
Assumption checks: StepTime_std.x
Model residuals
Shapiro−Wilk W = 0.963, p = 0.0325
Sample quantiles
Theoretical quantiles
0.004
0.002
0.000
−0.002
−2
−1
0
1
2
Subject random intercepts
Shapiro−Wilk W = 0.855, p = 0.0102
Sample quantiles
Theoretical quantiles
0.0075
0.0050
0.0025
0.0000
−0.0025
−0.0050
Homoscedasticity check
Spearman rho = 0.265, p = 0.0245
Absolute residuals
Fitted values
0.003
0.002
0.001
0.000
0.0075
0.0100
0.0125
0.0150
0.0175

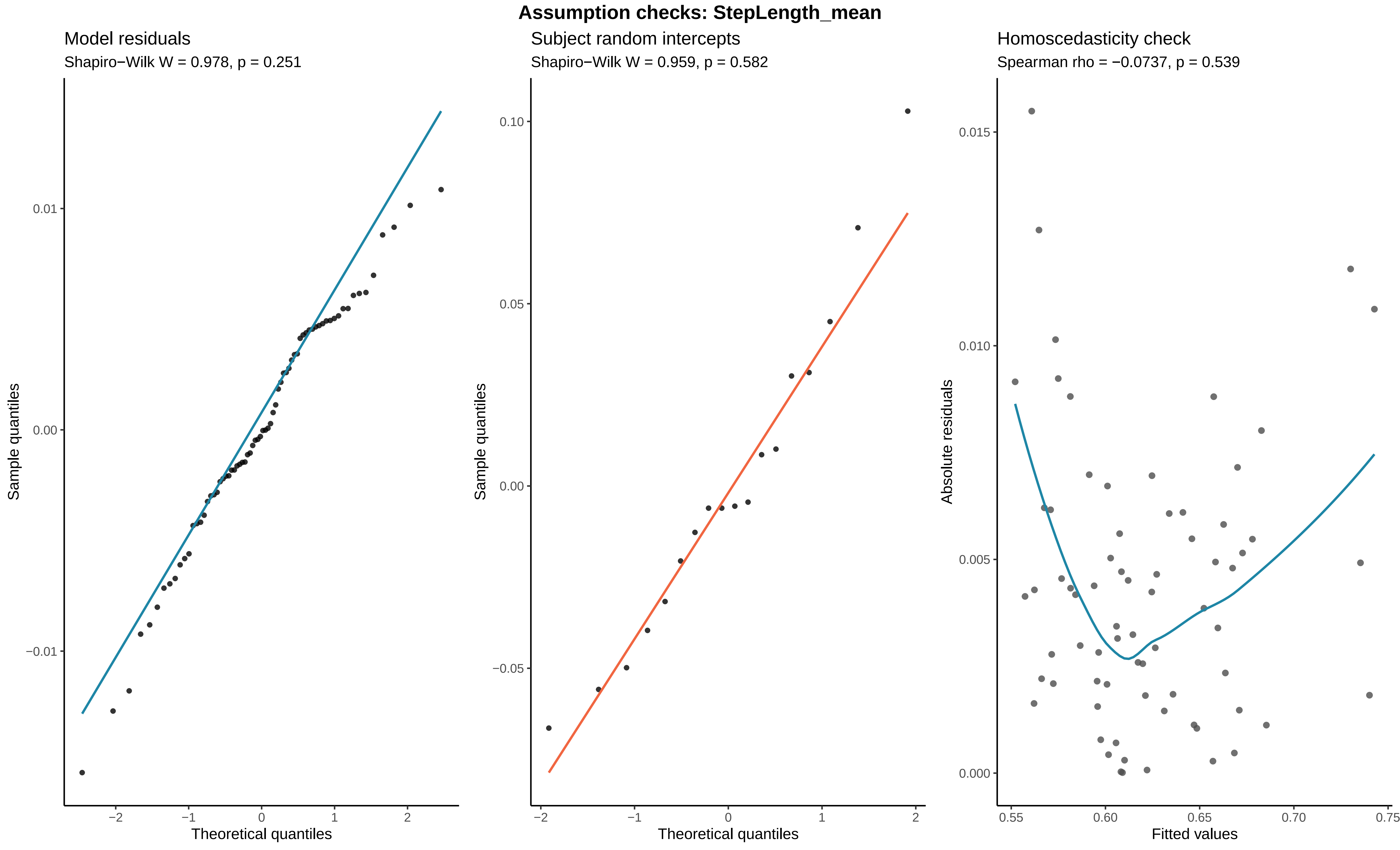

Assumption checks: StepLength_mean
Model residuals
Shapiro−Wilk W = 0.978, p = 0.251
Sample quantiles
Theoretical quantiles
Subject random intercepts
Shapiro−Wilk W = 0.959, p = 0.582
Sample quantiles
Theoretical quantiles
Homoscedasticity check
Spearman rho = −0.0737, p = 0.539
Absolute residuals
Fitted values

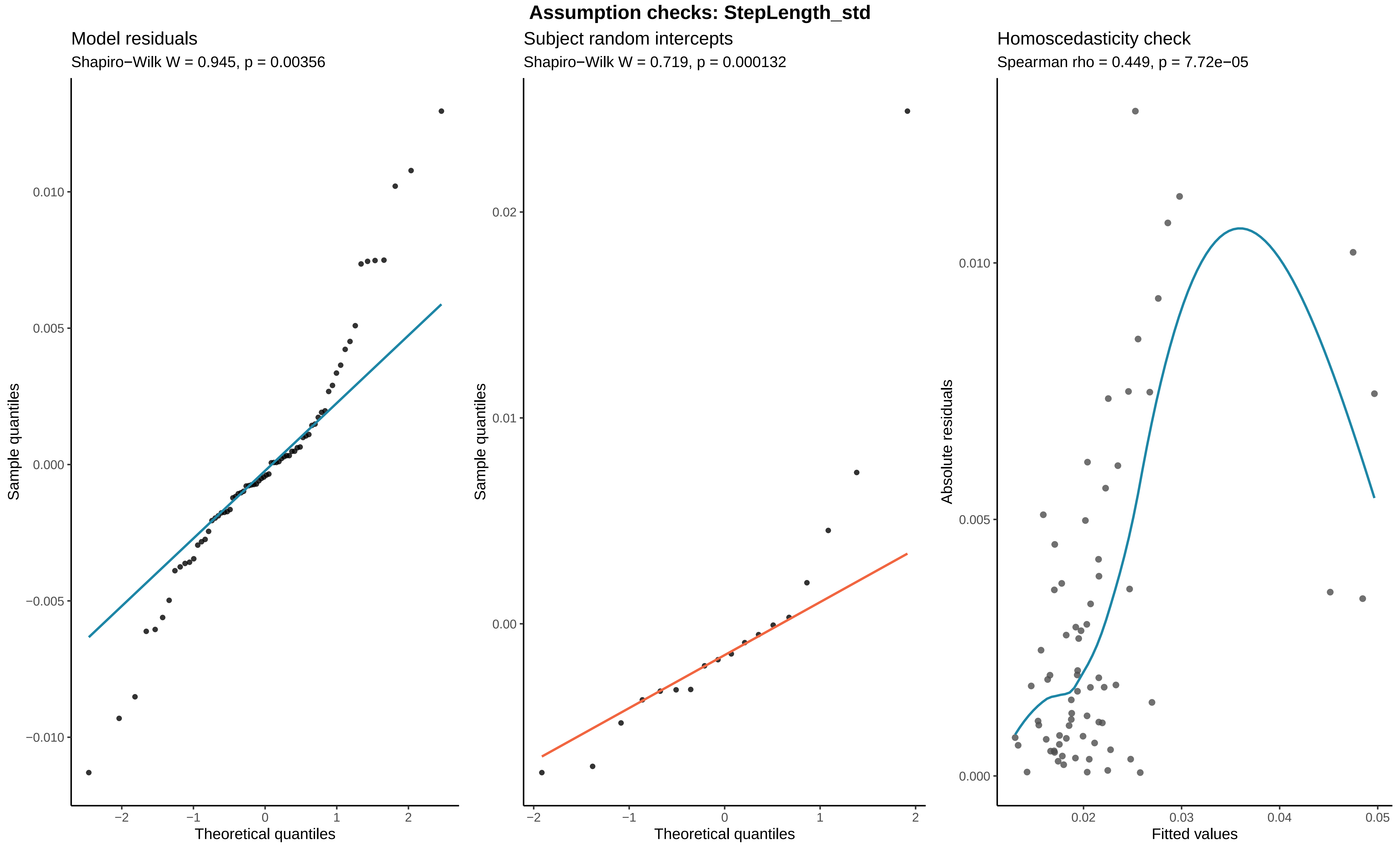

Assumption checks: StepLength_std
Model residuals
Shapiro−Wilk W = 0.945, p = 0.00356
Sample quantiles
Theoretical quantiles
0.010
0.005
0.000
−0.005
−0.010
−2
−1
0
1
2
Subject random intercepts
Shapiro−Wilk W = 0.719, p = 0.000132
Sample quantiles
Theoretical quantiles
0.02
0.01
0.00
Homoscedasticity check
Spearman rho = 0.449, p = 7.72e−05
Absolute residuals
Fitted values
0.010
0.005
0.000
0.02
0.03
0.04
0.05

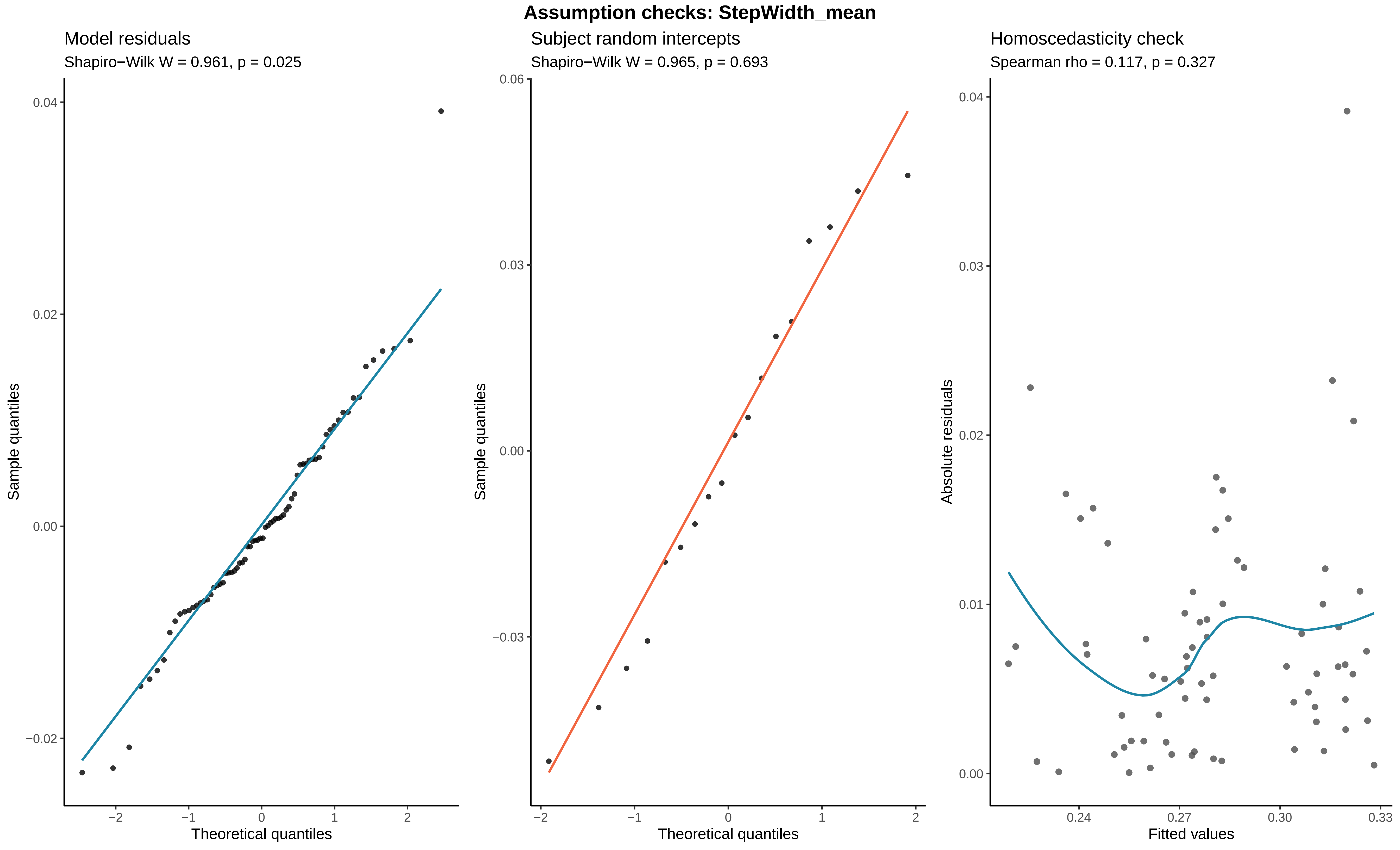

Assumption checks: StepWidth_mean
Model residuals
Shapiro−Wilk W = 0.961, p = 0.025
Sample quantiles
Theoretical quantiles
0.04
0.02
0.00
−0.02
−2
−1
0
1
2
Subject random intercepts
Shapiro−Wilk W = 0.965, p = 0.693
Sample quantiles
Theoretical quantiles
0.06
0.03
0.00
−0.03
Homoscedasticity check
Spearman rho = 0.117, p = 0.327
Absolute residuals
Fitted values
0.04
0.03
0.02
0.01
0.00
0.24
0.27
0.30
0.33

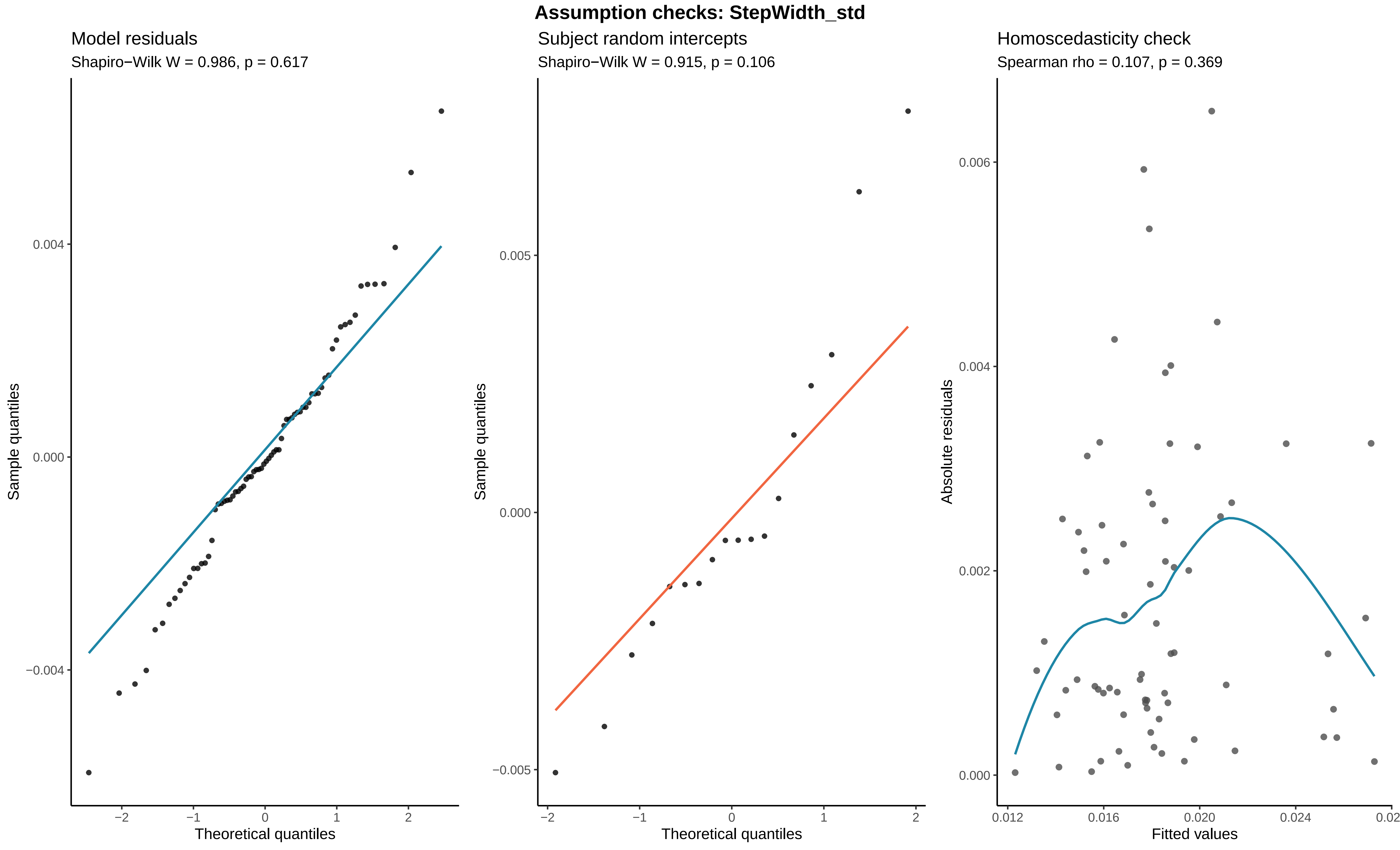
Assumption checks: StepWidth_std
Model residuals
Shapiro−Wilk W = 0.986, p = 0.617
Sample quantiles
Theoretical quantiles
0.004
0.000
−0.004
−2
−1
0
1
2
Subject random intercepts
Shapiro−Wilk W = 0.915, p = 0.106
Sample quantiles
Theoretical quantiles
0.005
0.000
−0.005
Homoscedasticity check
Spearman rho = 0.107, p = 0.369
Absolute residuals
Fitted values
0.006
0.004
0.002
0.000
0.012
0.016
0.020
0.024

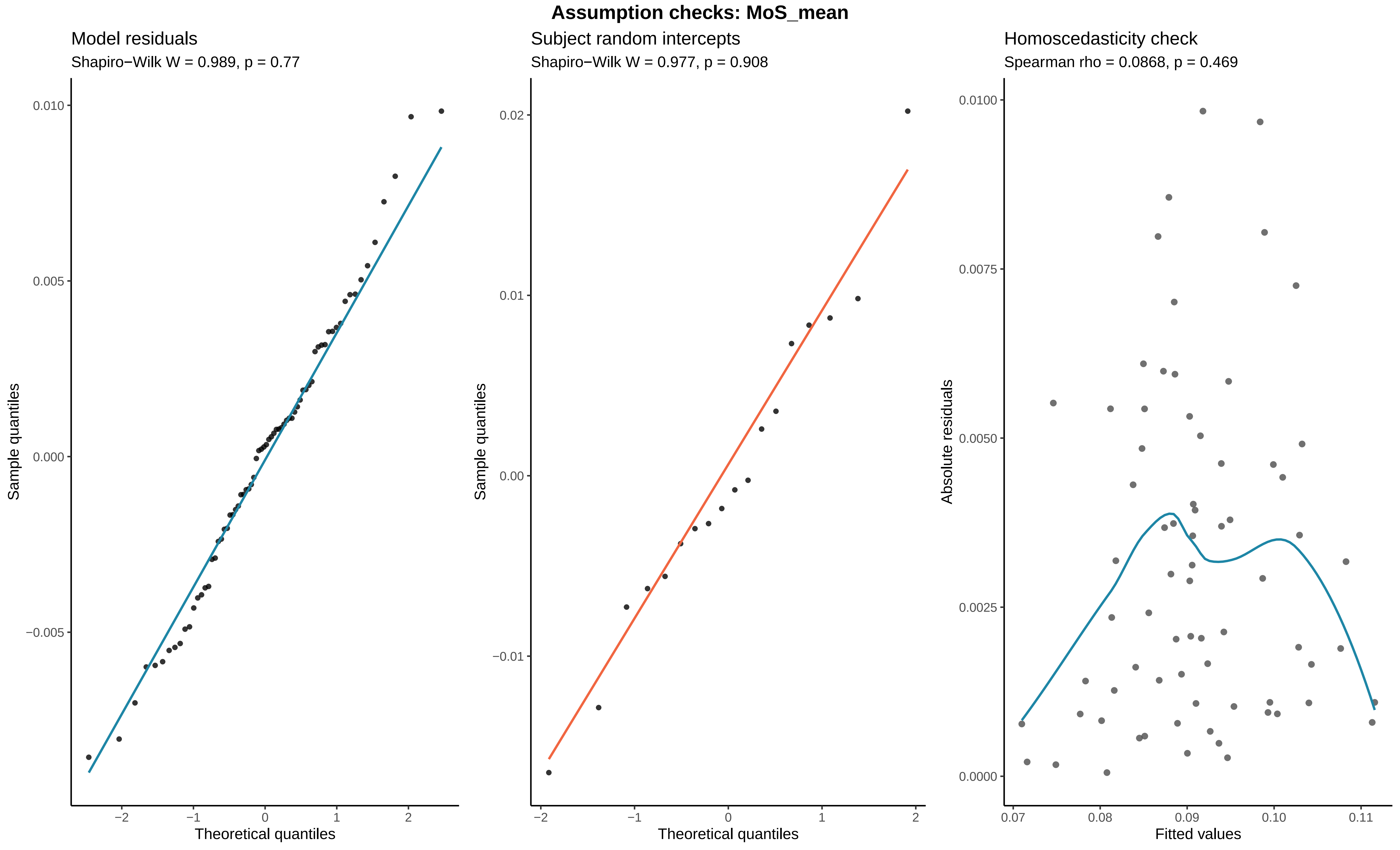
Assumption checks: MoS_mean
Model residuals
Shapiro−Wilk W = 0.989, p = 0.77
Sample quantiles
Theoretical quantiles
Subject random intercepts
Shapiro−Wilk W = 0.977, p = 0.908
Sample quantiles
Theoretical quantiles
Homoscedasticity check
Spearman rho = 0.0868, p = 0.469
Absolute residuals
Fitted values

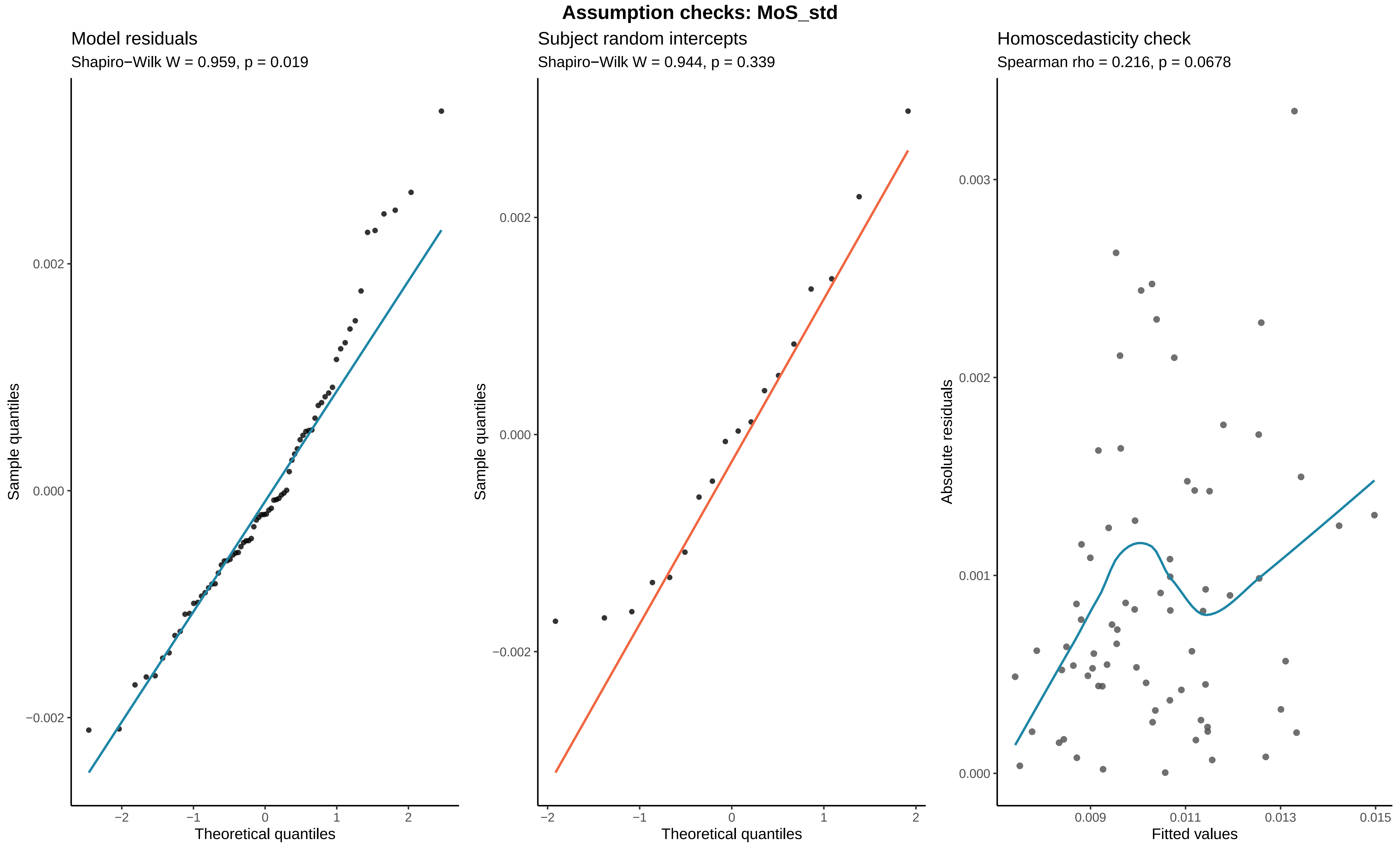

Assumption checks: MoS_std
Model residuals
Shapiro−Wilk W = 0.959, p = 0.019
Sample quantiles
Theoretical quantiles
0.002
0.000
−0.002
−2
−1
0
1
2
Subject random intercepts
Shapiro−Wilk W = 0.944, p = 0.339
Sample quantiles
Theoretical quantiles
0.002
0.000
−0.002
−2
−1
0
1
2
Homoscedasticity check
Spearman rho = 0.216, p = 0.0678
Absolute residuals
Fitted values
0.003
0.002
0.001
0.000
0.009
0.011
0.013
0.015

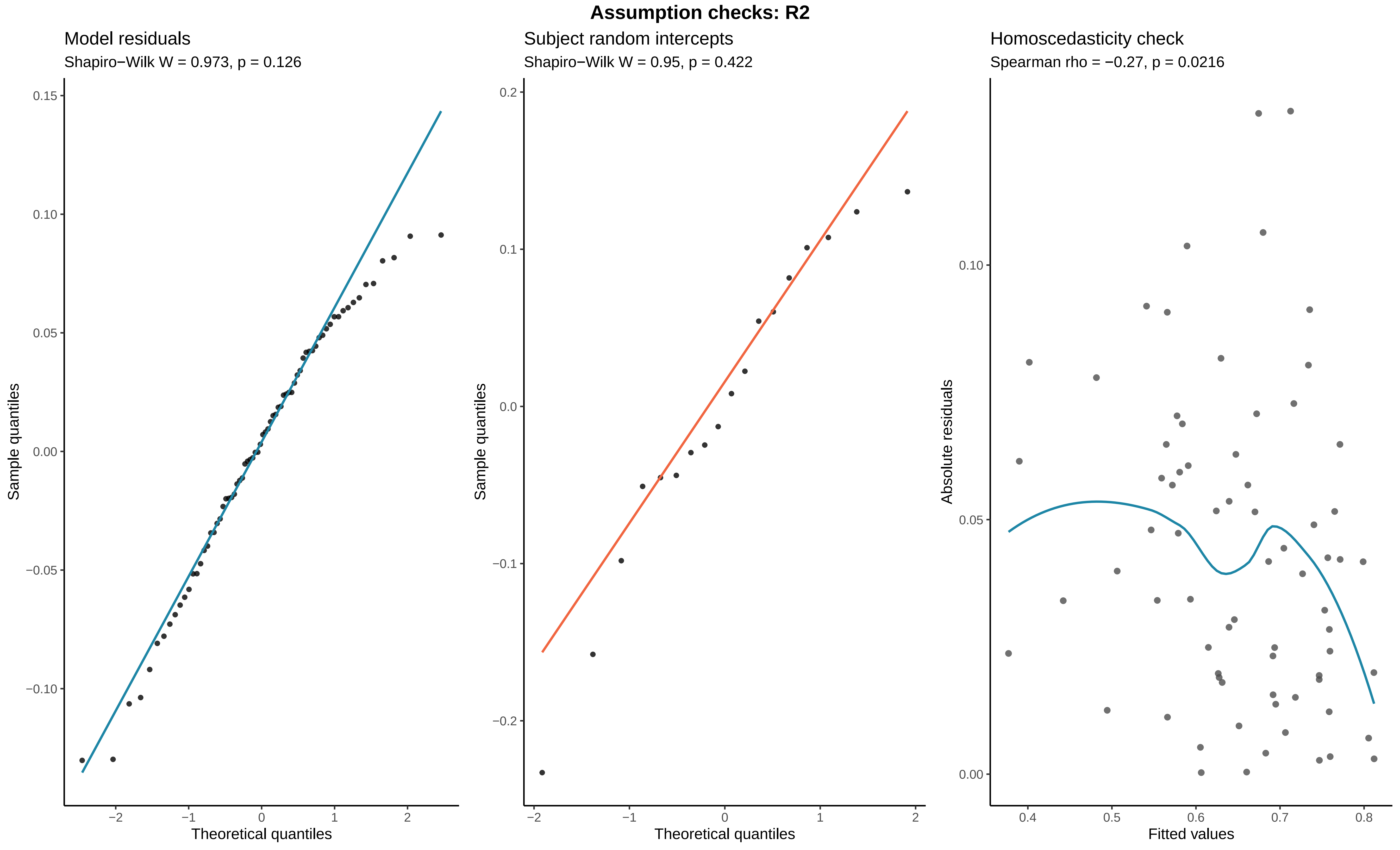

Assumption checks: R2
Model residuals
Shapiro−Wilk W = 0.973, p = 0.126
Sample quantiles
Theoretical quantiles
Subject random intercepts
Shapiro−Wilk W = 0.95, p = 0.422
Sample quantiles
Theoretical quantiles
Homoscedasticity check
Spearman rho = −0.27, p = 0.0216
Absolute residuals
Fitted values

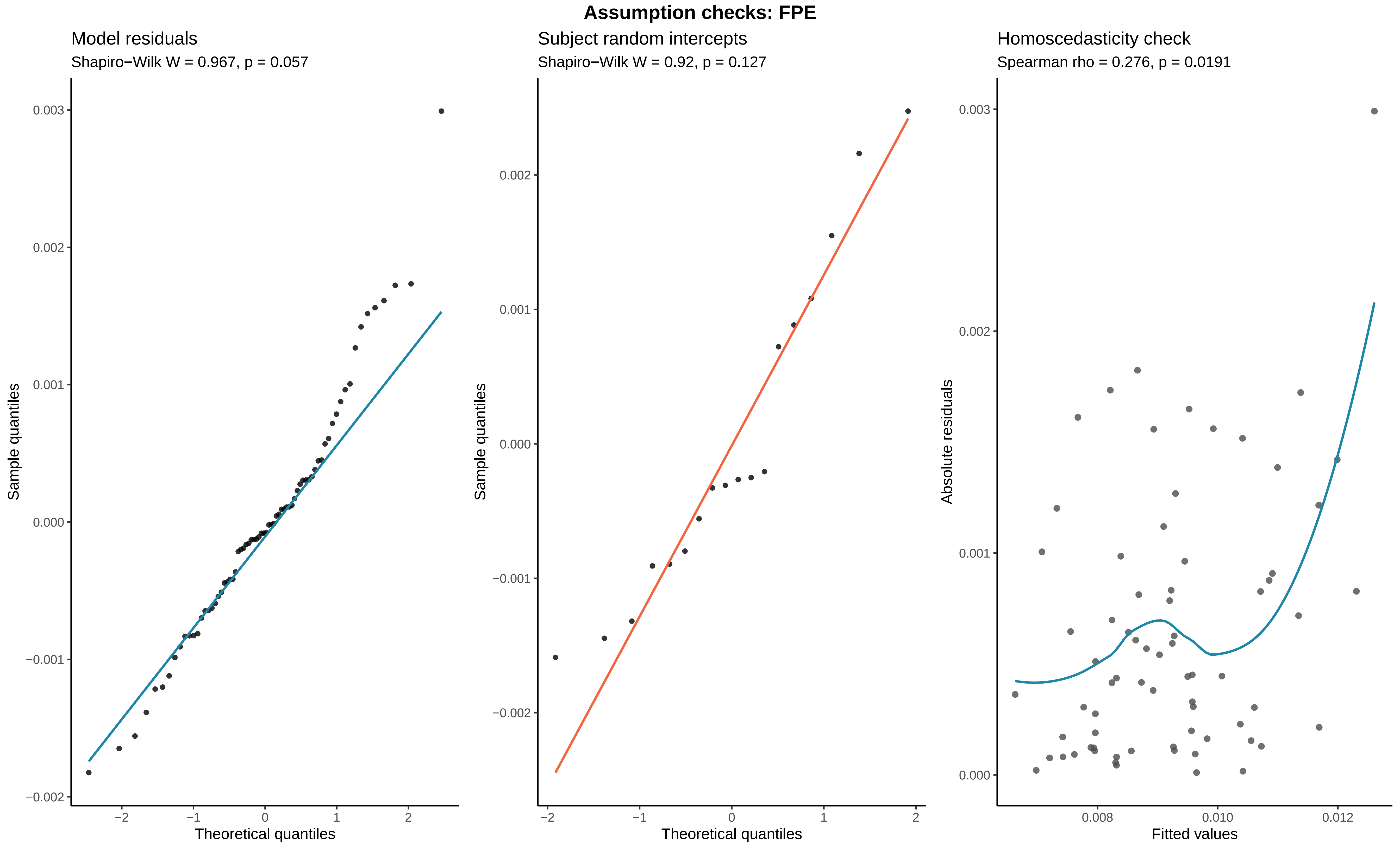
Assumption checks: FPE
Model residuals
Shapiro−Wilk W = 0.967, p = 0.057
Sample quantiles
Theoretical quantiles
0.003
0.002
0.001
0.000
−0.001
−0.002
−2
−1
0
1
2
Subject random intercepts
Shapiro−Wilk W = 0.92, p = 0.127
Sample quantiles
Theoretical quantiles
0.002
0.001
0.000
−0.001
−0.002
−2
−1
0
1
2
Homoscedasticity check
Spearman rho = 0.276, p = 0.0191
Absolute residuals
Fitted values
0.003
0.002
0.001
0.000
0.008
0.010
0.012

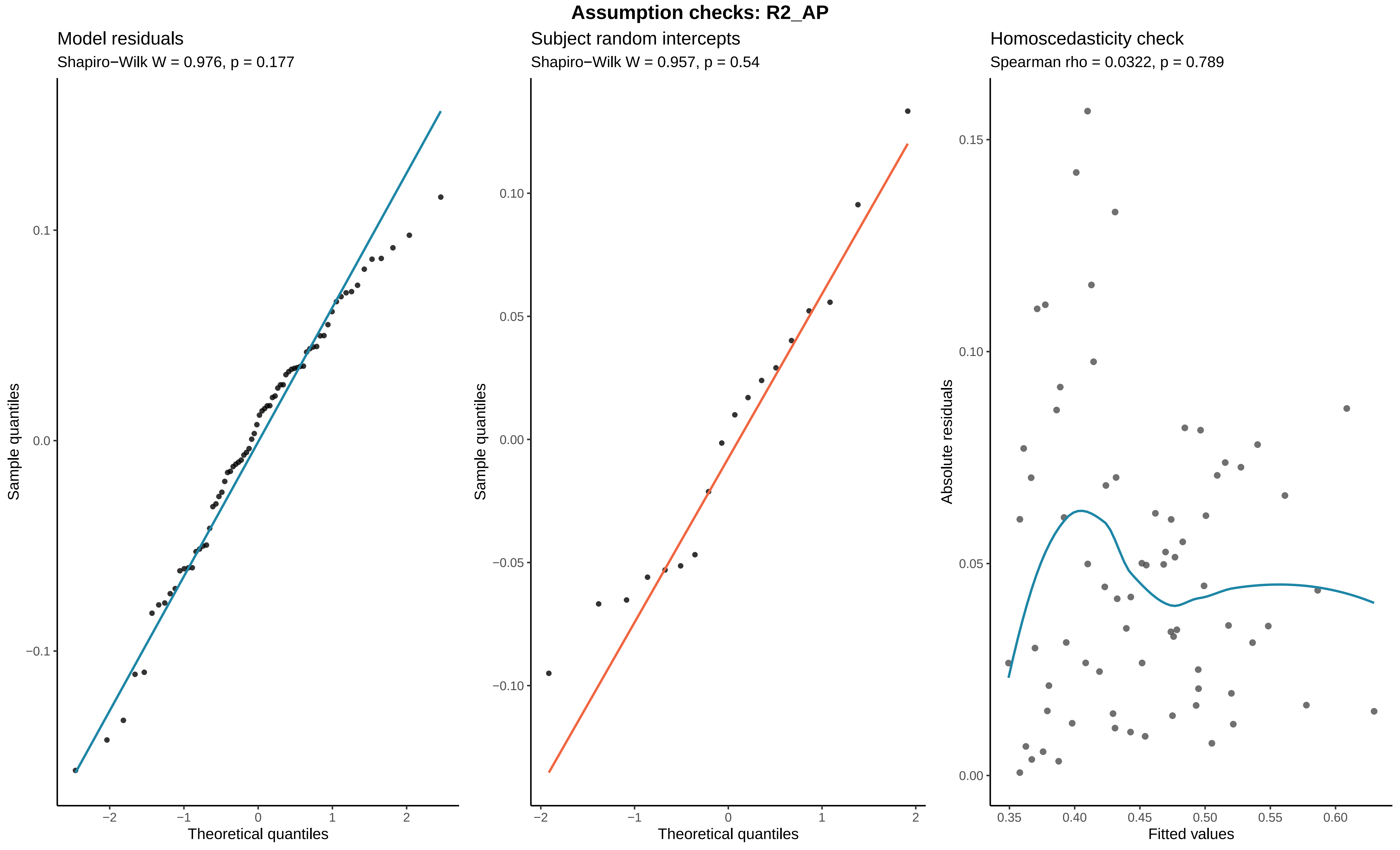

Assumption checks: R2_AP
Model residuals
Shapiro−Wilk W = 0.976, p = 0.177
Sample quantiles
Theoretical quantiles
Subject random intercepts
Shapiro−Wilk W = 0.957, p = 0.54
Sample quantiles
Theoretical quantiles
Homoscedasticity check
Spearman rho = 0.0322, p = 0.789
Absolute residuals
Fitted values

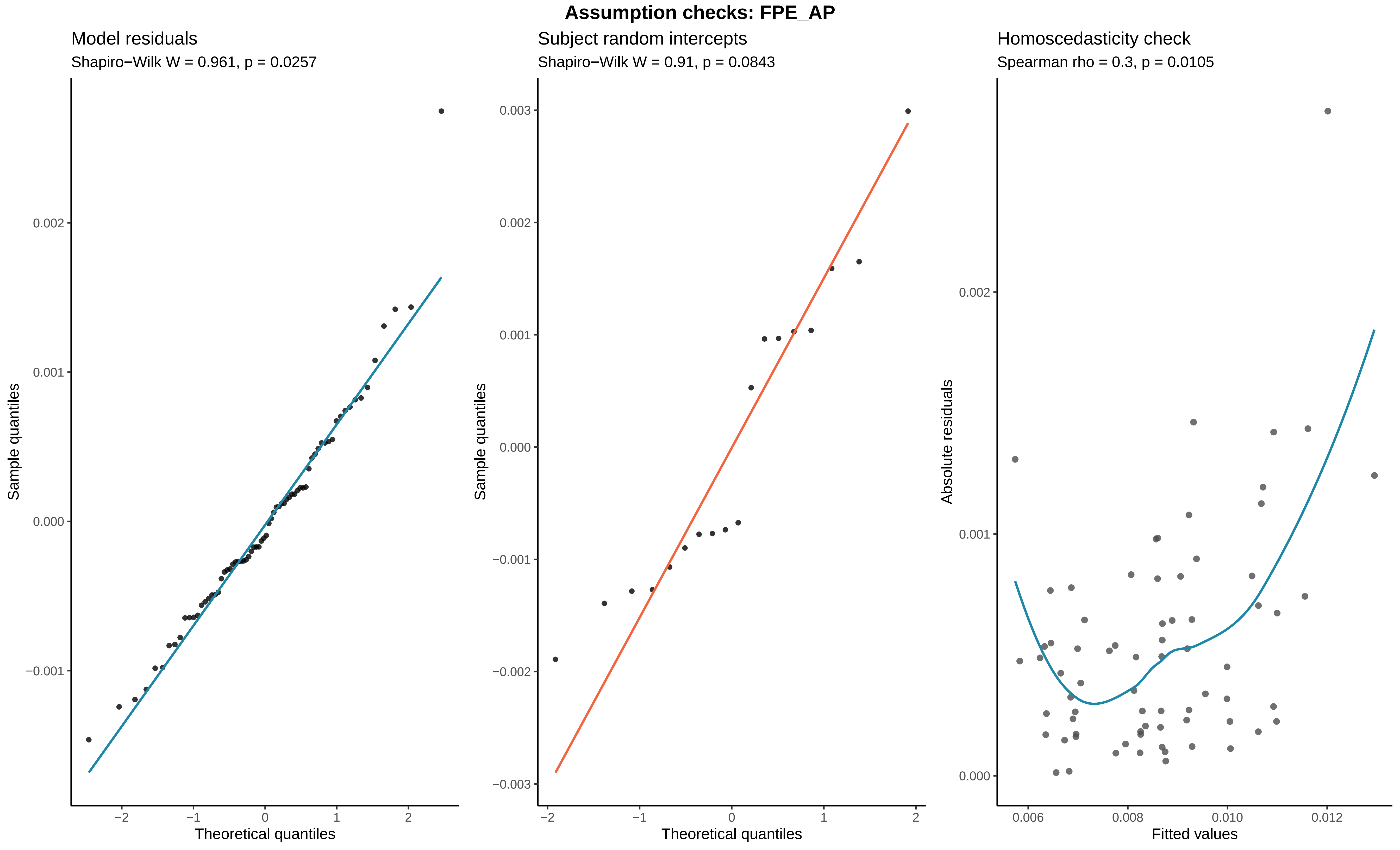
Assumption checks: FPE_AP
Model residuals
Shapiro−Wilk W = 0.961, p = 0.0257
Sample quantiles
Theoretical quantiles
Subject random intercepts
Shapiro−Wilk W = 0.91, p = 0.0843
Sample quantiles
Theoretical quantiles
Homoscedasticity check
Spearman rho = 0.3, p = 0.0105
Absolute residuals
Fitted values
